# Fit for purpose of on-the-road driving and simulated driving: A randomised crossover study using the effect of sleep deprivation

**DOI:** 10.1371/journal.pone.0278300

**Published:** 2023-02-02

**Authors:** Ingrid Koopmans, Robert-Jan Doll, Hein van der Wall, Marieke de Kam, Geert Jan Groeneveld, Adam Cohen, Rob Zuiker

**Affiliations:** 1 Centre for Human Drug Research, Leiden, The Netherlands; 2 Leiden University Medical Center, Leiden, The Netherlands; Charité - Universitätsmedizin Berlin, GERMANY

## Abstract

**Introduction:**

Drivers should be aware of possible impairing effects of alcohol, medicinal substance, or fatigue on driving performance. Such effects are assessed in clinical trials, including a driving task or related psychomotor tasks. However, a choice between predicting tasks must be made. Here, we compare driving performance with on-the-road driving, simulator driving, and psychomotor tasks using the effect of sleep deprivation.

**Method:**

This two-way cross over study included 24 healthy men with a minimum driving experience of 3000km per year. Psychomotor tasks, simulated driving, and on-the-road driving were assessed in the morning and the afternoon after a well-rested night and in the morning after a sleep-deprived night. Driving behaviour was examined by calculating the Standard Deviation of Lateral Position (SDLP).

**Results:**

SDLP increased after sleep deprivation for simulated (10cm, 95%CI:6.7–13.3) and on-the-road driving (2.8cm, 95%CI:1.9–3.7). The psychomotor test battery detected effects of sleep deprivation in almost all tasks. Correlation between on-the-road tests and simulator SDLP after a well-rested night (0.63, p < .001) was not present after a night of sleep deprivation (0.31, p = .18). Regarding the effect of sleep deprivation on the psychomotor test battery, only adaptive tracking correlated with the SDLP of the driving simulator (-0.50, p = .02). Other significant correlations were related to subjective VAS scores.

**Discussion:**

The lack of apparent correlations and difference in sensitivity of performance of the psychomotor tasks, simulated driving and, on-the-road driving indicates that the tasks may not be interchangeable and may assess different aspects of driving behaviour.

## Introduction

In the last decades, public and private organisations tried to improve automobile safety and decrease unsafe driving practices by addressing impaired driving [1]. Despite efforts, road trauma is still a significant public health issue [2]. A major cause of driving crashes and deaths is drowsy driving and/or driving under influence [3, 4]. This has recently been confirmed in a systematic review and meta-analysis in which a strong association between sleepiness and car accidents was established [5]. Additionally, several research groups demonstrated that sleep deprivation impairs driving performance in simulated driving and on-the-road driving tests.

Driving performance must be captured in a reliable, repeatable, and sensitive manner to study the effect of interventions (e.g., sleep deprivation, medication, or distractions in the car). Limiting the environmental variables (e.g., interaction with other road users) and standardising road conditions (e.g., length, number of lanes, or speed limit) supports the creation of a standardised test. Many on-the-road driving studies are performed on a highway where subjects stay in a single lane and thus limit their interactions with other drivers. While this standardised procedure results in reliable and repeatable results, one could argue that this costly and time-consuming measurement can be easily replaced with a driving simulator.

Simulated driving is a widely used alternative for on-the-road driving. Besides cost-effectiveness, high levels of drugs and alcohol can be tested in a laboratory setting with medical assistance nearby. This makes the driving simulator an attractive and safer method to study healthcare interventions on driving behaviour. Moreover, in 2017, the United States Food and Drug Administration (FDA) started accepting driving simulator studies for the registration of (new) drugs in some conditions [6]. However, as there is a wide variety in the validity of simulators ranging from simple single-monitor desk setups to hydraulic based widescreen setups, it is known that the driving experience in a simulator can be considered unrealistic. The lack of consequences (e.g., after a car crash) might preclude a sense of fear and vigilance, resulting in a distorted representation of the drug effect. Even though simulators are not always found to have similar sensitivity to drug effects as the on-the-road task [7, 8], driving simulators can detect impaired driving performance induced by sleep deprivation and well-known drugs [9–11].

Driving performance is often quantified by measuring a single measure describing the swaying/waving of the car and expressed with the standard deviation of the lateral position (SDLP). This measure is sensitive to CNS modulators (e.g., sleep deprivation, alcohol, and drugs [12–14]), and is regarded as reflecting overall driving [15]. However, when testing skills in isolation, such as hand-eye coordination, concentration, and decision making, correlations for these individual tests with the SDLP are modest at the most [9, 13, 16]. Therefore, it remains intriguing to better understand the contribution of cognitive domains to driving performance. Integrating isolated skills with those derived from the driving task could provide detailed information on intervention effects. In fact, combining cognitive/motor performance, on-the-road driving, and simulated driving in a single study, is rarely explored. Here, we aim to compare the impact of sleep deprivation on driving performance using on-the-road driving, simulator driving, and psychomotor tasks in healthy subjects.

## Methods

This was a single-centre randomised, two-way cross-over study. The study was conducted at the clinical research unit of the Centre for Human Drug Research (CHDR) in Leiden, The Netherlands. The study was approved by the Medical Ethics Committee Stichting Beoordeling Ethiek Biomedisch Onderzoek (Assen, the Netherlands) and registered under NL68626.056.19. The study was conducted according to the Dutch Act on Medical Research Involving Human Subjects (WMO) and in compliance with all International Conference on Harmonisation Good Clinical Practice (ICH-GCP) guidelines.

### Participants

**Fig 1:**
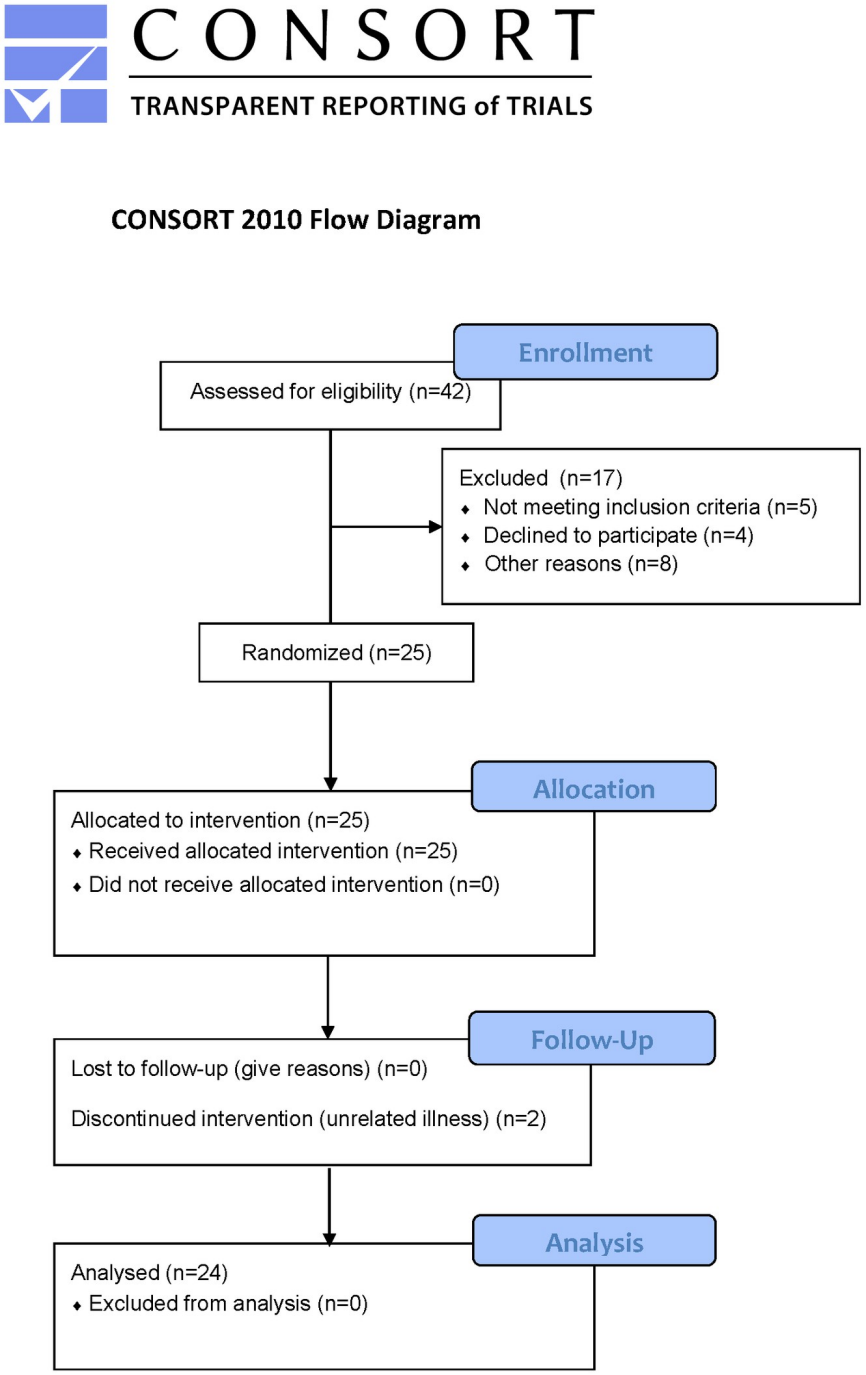
CONSORT flow chart of screening, participation and analysis.

All participants provided written informed consent before screening and study-related activities. This study was a part of a more extensive research on the effects of sleep deprivation> Next to the impact of sleep deprivation on driving, effects on pain thresholds were assessed. Because of the influence of the ovarian cycle on pain thresholds, initially, only men were invited to participate. For the latter assessment, only men between 23 and 35 years of age were invited to participate in the study. Only experienced drivers, defined as participants having a valid driving licence for at least five years and having, on average, a self-reported annual mileage of at least 3000 km, were included in the study. No history or presence of sleep disorders was allowed. Participants had to remain in the same time zone as the Netherlands at least seven days before the first visit and during the trial period. Additionally, participants showing signs of Simulator Sickness Syndrome before or during a screening of the simulated driving sessions were not eligible to participate. See Fig 1 for the CONSORT flow diagram.

### Experiment design

**Fig 2:**
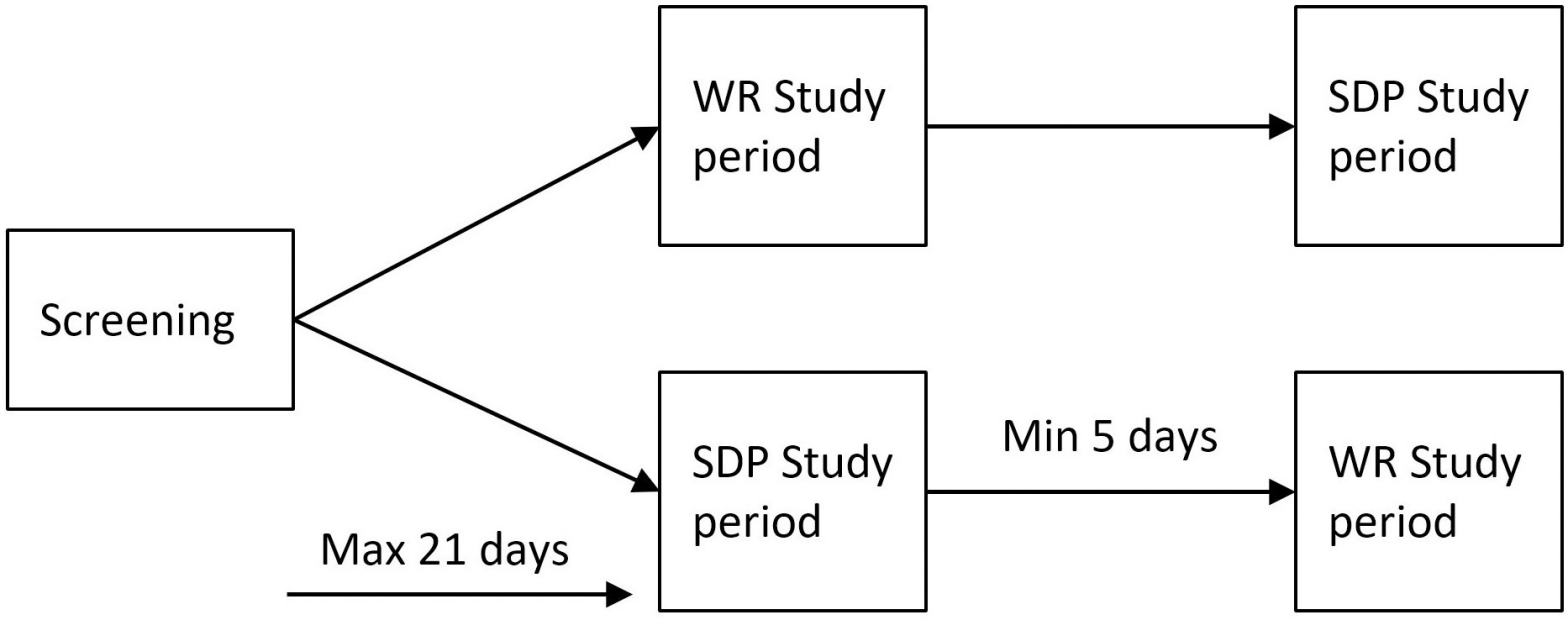
Schematic overview of the study design. WR: Well-rested. SDP: Sleep Deprived.

During the screening period, all participants were trained on the study assessments by completing the full or a shortened version of each test. After inclusion, participants attended the clinic on two visits: a sleep-deprivation visit, and a well-rested visit. Participants were randomized on the order of visits, with at least 5 days to recover from the sleep deprivation if performed first (see Fig 2). Participants were not allowed to consume caffeine, alcohol or drugs during the clinical trial starting 4, 24 hours and 3 days prior to the first visit, respectively. To make sure the participants were well rested prior to the study, they were asked to maintain a normal sleep rhythm (at least 8 hours between 22:00 and 8:00) for two nights prior to the visits. For the sleep deprived (SDP) visit, participants arrived in the late afternoon and could leave the day after. Participants were kept awake throughout the night after arrival. All assessments were performed in the morning after sleep deprivation. During the visit, participants were allowed only light physical activities (e.g., foosball). During the well-rested (WR) visit, participants were instructed to sleep at home for at least 8 hours before they were admitted to the clinical unit. All assessments were subsequently completed twice: once in the morning and once in the afternoon.

### Assessments

**Fig 3:**
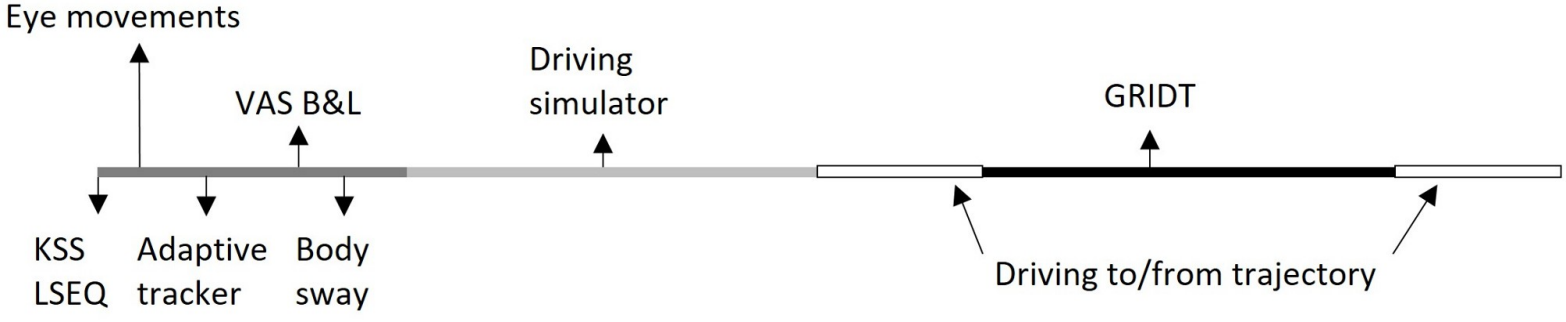
Schematic overview of the order of tests.

The assessments contain simulated driving, on-the-road driving, the performance of a cognitive test battery (NeuroCart), and general questionnaires. The cognitive test battery consists of six tests: eye movement test (both smooth pursuit and saccadic), adaptive tracker, VAS Bond & Lader, Karolinska Sleepiness Scale and the body sway test. All measurements were performed in a quiet room with dimmed lightning. There was only one subject per session in the same room. The order of the assessments for each round of assessments is visualized in Fig 3.

#### On-the-road driving

**Fig 4:**
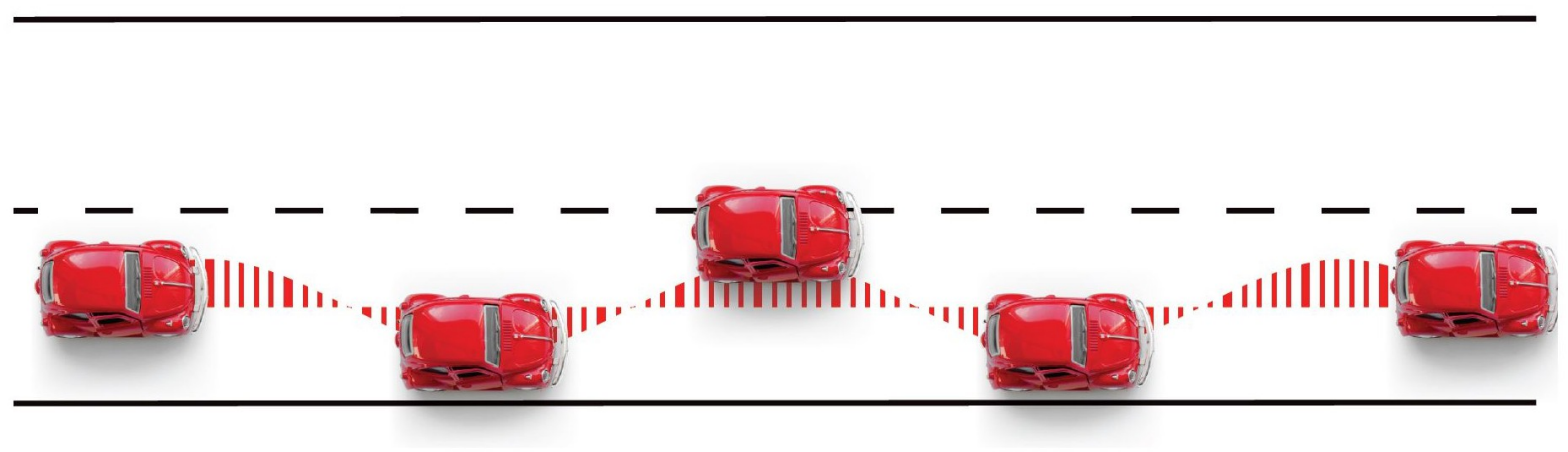
Visual impression of the car weaving within a lane. The Standard Deviation of Lateral Position (SDLP) is calculated as the standard deviation of the sway around the average position within the lane.

For on-the-road driving, a car (Volkswagen caddy) was specifically modified with safety and measurement equipment (GRIDT). The location of the car was recorded using a GPS sensor mounted on the roof of the car. A Mobileye system (MobilEye Vision Technologies Ltd., Israel) was used to determine the relative position of the car on the road and log the speedometer (both sampled with a frequency of 13Hz). This data was used to determine the Standard Deviation of Lateral Position (SDLP) of the driving session [17]. See Fig 4 for a visual impression of the SDLP. For safety reasons, a certified driving instructor sat on the passenger seat during all on-the-road assessments and had access to dual controls. Subjects were instructed to drive on a predefined section of a public road (N11, the Netherlands) and to maintain a steady speed of 95km/h. Subjects were instructed to only overtake other vehicles when this was required for maintaining a steady speed. The chosen trajectory was a 40 km long two-lane highway starting at 15-minute drive from the clinical unit with a speed limit of 100 km/h. The road contained two sections including traffic lights with a speed limit of 70km/h for 0.5 km, each. Subjects were to drive on this road in both directions turning the vehicle after 26km. The subjects received instructions identical to the published SOP by Verster et al. [15] prior to the start of the drive and these instructions were repeated on request. A research assistant was present in the car to operate the data logging system.

**Fig 5:**
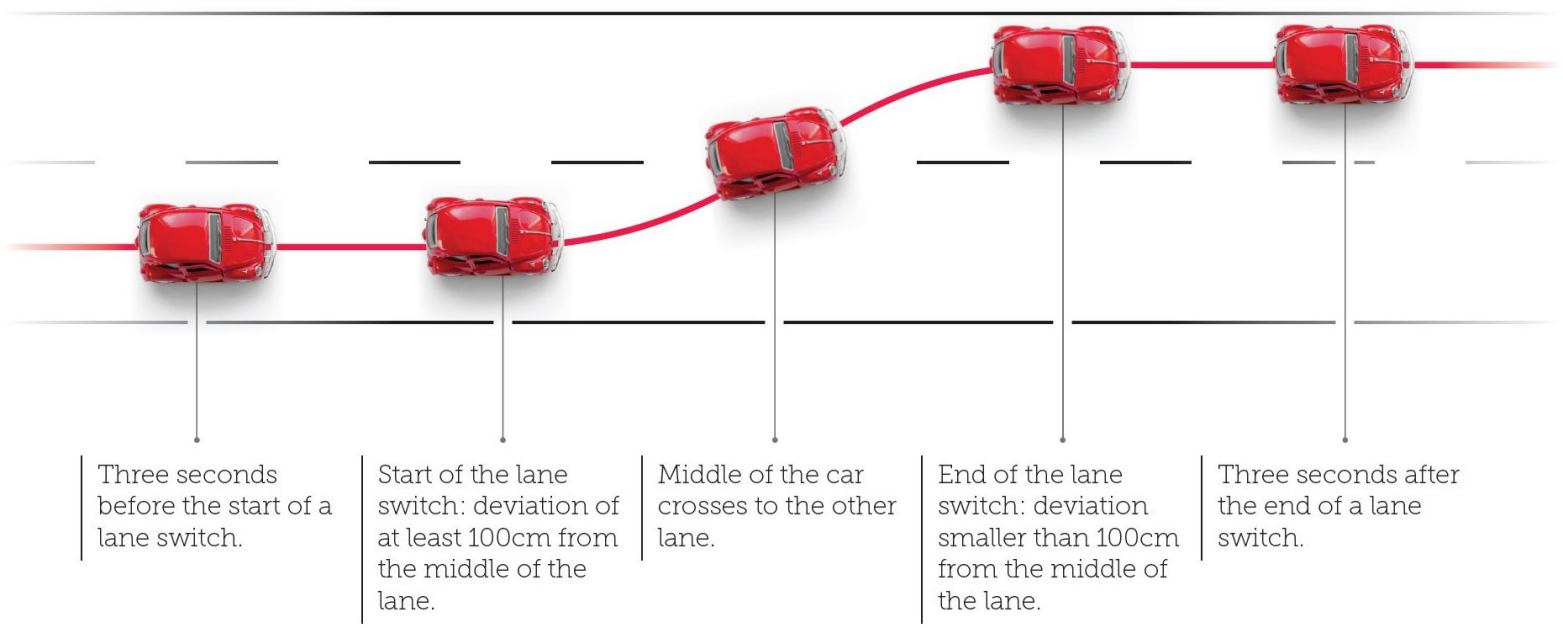
Schematic overview of data removal during a lane switch. All data between three seconds before until three seconds after a lane switch is removed prior to analysis.

*Cleaning of the on-the-road data*. Data outside the trajectory of interest was removed using the GPS prior to data analysis. Additionally, measurements outside the speed range of 85–110 km/h and during successful lane switches were excluded from the analysis data set. A successful lane switch is defined as the crossing of the white stripes in the road with the middle of the car. The start of a lane switch is a deviation of the middle lane of at least 100cm. The end of a lane switch is defined as the moment when the car is within 100cm of the middle of the new lane. To make sure pre-lane and post-lane switch behaviour (e.g., the intention to switch lanes) is also excluded from the data, three seconds before the start and after the end of a lane switch is included in the removal of data. Therefore, the data collected between two-lane switches (i.e., to the left lane and back) can be kept for analysis. The data removal is illustrated in Fig 5.

#### Simulated driving

The simulated driving test was performed on a fix-based driving simulator (Drivemaster, Green Dino B.V., the Netherlands) [9, 18]. Each experiment session lasted for 20 minutes of driving on a two-lane highway with traffic. Subjects were instructed to maintain a steady speed of 100 km/h on the outer lane. Overtaking was only allowed for maintaining a steady speed. The first five minutes were removed prior to data analysis [19]. A test drive of 15 minutes was performed on the same simulated highway trajectory during screening. Lane switches were removed following the same procedure as described above.

#### Driving questionnaires

After each (simulated) driving task a combined perceived driving effort and quality scale was used to record a self-assessment of the subjects driving performance [15]. The performance and motivation score is a VAS scale running from 1 (worst driving performance possible) to 15 (best driving performance possible). The perceived effort scale is a labelled VAS scale with 1 (no effort at all) and 15 (most effort possible). The driving instructor provided an opinion on subjects driving behaviour on the road with a scale of 1 to 10, with 10 representing perfect driving behaviour, on 11 aspects of driving: scanning, change of gear, steering, breaking, use of clutch, speed, rounding corners, anticipation on surroundings, applying traffic regulations, attention and reaction time. The total score was used for further analysis.

#### Eye movement measurements

Recording and analysis of saccadic eye movements is conducted with a microcomputer-based system that samples and analyses eye movements. The program for signal collection and the AD-converter is from Cambridge Electronic Design (CED Ltd., Cambridge, UK), the signal amplification using Grass (Grass-Telefactor, An Astro-Med, Inc. Product Group, Braintree, USA) and the sampling and analysis scripts are developed at the CHDR (Leiden, the Netherlands). Disposable silver-silver chloride electrodes (Ambu Blue Sensor N) will be applied on the forehead and beside the lateral canthi of both eyes of the subject for registration of the electro-oculographic signals. Skin resistance is reduced to less than 5 kOhm before measurements by scrubbing the skin and using electrolyte gel. Head movements are restrained using a fixed head support. The target consists of a moving dot that is displayed on a computer screen. This screen is fixed at 58 cm in front of the head support.

Saccadic eye movements are recorded for approximately 15 degrees to either side for stimulus amplitudes. Fifteen saccades are recorded with interstimulus intervals varying randomly between 3 and 6 seconds. Average values of latency (reaction time), saccadic peak velocity of all correct saccades and inaccuracy of all saccades will be used as parameters. Saccadic inaccuracy is calculated as the absolute value of the difference between the stimulus angle and the corresponding saccade, expressed as a percentage of the stimulus angle. Saccadic peak velocity is one of the most sensitive parameters for sedation [20, 21]. The use of a computer for measurement of saccadic eye movements was originally described by Baloh et al. [22], and has been validated at CHDR by Van Steveninck et al. [20].

For smooth pursuit eye movements, the target moves at a frequency ranging from 0.3 to 1.1 Hz, by steps of 0.1 Hz. The amplitude of target displacement corresponds to 22.5 degrees eyeball rotation to both sides. Four cycles are recorded for each stimulus frequency. The time in which the eyes are in smooth pursuit of the target will be calculated for each frequency and expressed as a percentage of stimulus duration. The average percentage of smooth pursuit for all stimulus frequencies will be used as parameter. The method has been validated at CHDR by Van Steveninck et al. [23, 24] based on the work of Bittencourt et al. [25] and the original description of Baloh et al. [26].

#### Adaptive tracker

The adaptive tracking test was performed using customised equipment and software (based on TrackerUSB hard-/software (Hobbs, 2004, Hertfordshire, UK)). This 3.5-minute period is including a run-in time of 0.5 minute, in this run-in time the data is not recorded. Adaptive tracking is a pursuit-tracking task. A circle moves randomly about a screen. The subject must try to keep a dot inside the moving circle by operating a joystick. If this effort is successful, the speed of the moving circle increases. Conversely, the velocity is reduced if the test subject cannot maintain the dot inside the circle. The average performance and the standard deviation of scores over 3.5 minutes will be used for analysis. The adaptive tracking test has proved sensitive for measurement of CNS effects of alcohol [27], various pharmacological compounds [28] and sleep deprivation [29].

#### Body sway

The body sway meter allows measurement of body movements in a single plane, providing a measure of postural stability. Body sway is measured with a pot string meter (Celesco) based on the Wright ataxiameter [30]. With a string attached to the waist, all body movements are integrated and expressed as mm sway. Before starting a measurement, subjects were asked to stand still and comfortable, with their feet approximately 10 cm part and their hands in a relaxed position alongside the body and eyes closed. The total sway during two minutes is used as a parameter for body sway. The method has been used to demonstrate effects of sleep deprivation [31], alcohol [32] and several pharmacological compounds [28].

#### VAS Bond & Lader

Visual analogue scales as originally described by Norris have often been used previously to quantify subjective effects of a variety of sedative agents [33, 34]. Subjects indicate (with a mouse click on the computer screen) on sixteen horizontal visual analogue scales how he feels. From these measurements, three main factors are the calculated as described by Bond and Lader [35]: alertness (from nine scores), contentedness (often called mood; from five scores), and calmness (from two scores).

#### Karolinska Sleepiness Scale (KSS)

The Karolinska Sleepiness Scale (KSS) [36] measures the participant’s state of sleepiness at a given moment in time. Participants were asked: ‘Use the following scale to indicate how sleepy you are feeling at this moment. Write the number in the box.’ Nine numerical response alternatives are listed vertically with verbal labels assigned to alternate numbers: 1. Extremely Alert; 2; 3 Alert; 4; 5 Neither Alert nor Sleepy; 6; 7 Sleepy But Not Fighting Sleep; 8; 9 Extremely Sleepy, Fighting Sleep, Effort to Stay Awake.

### Statistical analysis

All statistical analyses were performed using SAS version 9.4 (SAS Institute, Inc., Cary, NC, USA). A sample size calculation was performed using previous results of the simulator with a two-sided paired t-test [9]. A total sample size of n = 20 would be sufficient to determine significant differences in SDLP measured with the driving simulator of 2.5cm with a significance level of 0.05 and a power of 0.80. Accounting for technical malfunctions and subjects dropping out, we aimed to include 24 subjects.

Each variable was analysed with a mixed model analysis of variance with fixed factor condition (separate for each set of assessments) and random factor subject. Simulator mean speed, simulator standard deviation speed, GRIDT mean speed, and body sway were log-transformed to correct for a log-normal distribution before statistical analysis.

The repeatability was quantified by the coefficient of variation (CoV) within and between subjects, as estimated from the subject (between-subject) variability and residual (within-subject) variability of the mixed model analysis and the mean over the three conditions of the estimated least square means. The common variance is the sum of the inter and intrasubject variability. For log-transformed variables the CoV is calculated from the same estimated variabilities of the mixed model analyses which are back transformed by $100\;*\;\sqrt{e^{variability}-1}$ to a CoV.

Pearson correlations were calculated for the SDLP and each variable in each condition (well rested and sleep deprived). In case of log normal distribution of a variable the log values of the variable are used.

## Results

### Participants

A total of 25 participants were enrolled in the study from March to June 2019. Two subjects stopped participation during the night of sleep deprivation due to illness unrelated to the sleep deprivation. Because one of these subjects had sleep deprivation as his first visit, the subsequent well-rested visit was also not performed. Twenty-four subjects are included for statistical analysis (age mean (SD) is 25.7 (1.6) years, BMI is 24.3 (3.4) kg/m^2^). Data could not be collected during 3 GRIDT assessments (two during the sleep-deprivation visit and one afternoon session during the well-rested visit) due to technical difficulties.

### Repeatability of driving parameters

#### GRIDT

The repeatability of the GRIDT driving parameters (i.e., SDLP, mean speed, SD-speed) during the well-rested visit (morning and afternoon) is presented in Table 1. Results of driving parameters are presented in Table 2. The mean (SD) SDLP for the GRIDT was 21.33 cm (2.3) and 22.26 cm (2.4) during the morning and afternoon, respectively. The coefficient of variation (CoV) was 8.9% and 6.5% for the inter-and intra-subject variability, respectively. The common variance was 6.13 (CoV: 11.0%).

**Table 1:** Inter-subject, intra-subject, common variance, and minimal detectable effect size (MDES), calculated at the well-rested visit. CoV: Coefficient of Variation.

Variable		Inter-subject Variance (CoV)	Intra-subject Variance (CoV)	Common Variance (CoV)	MDES N = 16 cross-over
SDLP (cm)	Simulator	16.32 (11.8%)	31.67 (16.5%)	47.99 (20.3%)	6.0
	GRIDT	3.99 (8.9%)	2.14 (6.5%)	6.13 (11.0%)	1.6
Mean speed^1^ (km/h)	Simulator	1.2%	1.4%	1.9%	1.5%
	GRIDT	1.2%	1.0%	1.6%	1.1%
SD-speed^1^ (km/h)	Simulator	25.6%	21.3%	33.7%	25.0%
	GRIDT	14.7%	9.9%	17.8%	11.0%

^1^ Geometric mean based on logarithmic transformed data

**Table 2:** Results of driving-related parameters for both the simulator and GRIDT.

Parameter	SDLP [cm]		Mean Speed (km/h)		SD Speed (km/h)		Performance and motivation (cm)		Driving effort (cm)		Instructor assessment
	Simulator	GRIDT	Simulator^1^	GRIDT^1^	Simulator^1^	GRIDT^1^	Simulator	GRIDT	Simulator	GRIDT	GRIDT
Well rested morning (SD)	30.14 (5.2)	21.33 (2.3)	96.96 (1.6)	95.72 (1.4)	2.57(0.9)	3.55 (0.7)	8.4 (1.8)	9.1 (2.1)	3.4 (2.1)	3.3 (1.6)	67.0 (5.5)
Well rested afternoon (SD)	32.16 (5.5)	22.26 (2.4)	96.92 (1.9)	95.64 (1.4)	2.65 (1.0)	3.59 (0.7)	7.9 (2.2)	8.4 (2.4)	4.6 (2.9)	3.4 (2.2)	66.2 (6.3)
Sleep deprived (SD)	40.26 (9.4)	24.08 (2.7)	97.33 (1.9)	94.99 (1.7)	3.36 (1.1)	3.73 (0.5)	4.6(2.6)	5.6 (2.4)	10.3 (3.4)	9.2 (4.4)	61.1 (6.6)
Contrasts^2^ [95% CI]	9.97 (6.65–13.29)	2.76 (1.87, 3.66)	0.4% (-0.4%, 1.3%)	-0.7% (-1.3%, -0.1%)	28.7% (13.7%, 45.7%)	5.0% (-1.1%, 11.6%)	-3.8 (-5.0, -2.6)	-3.5 (-4.8, -2.2)	6.9 (5.7, 8.1)	5.9 (4.4, 7.4)	-5.9(-8.1, -3.7)
p-value^2^	< .001	< .001	0.34	< 0.05	< .001	0.11	< .001	< .001	< .001	< .001	< .001

^1^ Geometric mean based on logarithmic transformed data

^2^ Contrasts between the well-rested morning and sleep deprived

#### Driving simulator

**Fig 6:**
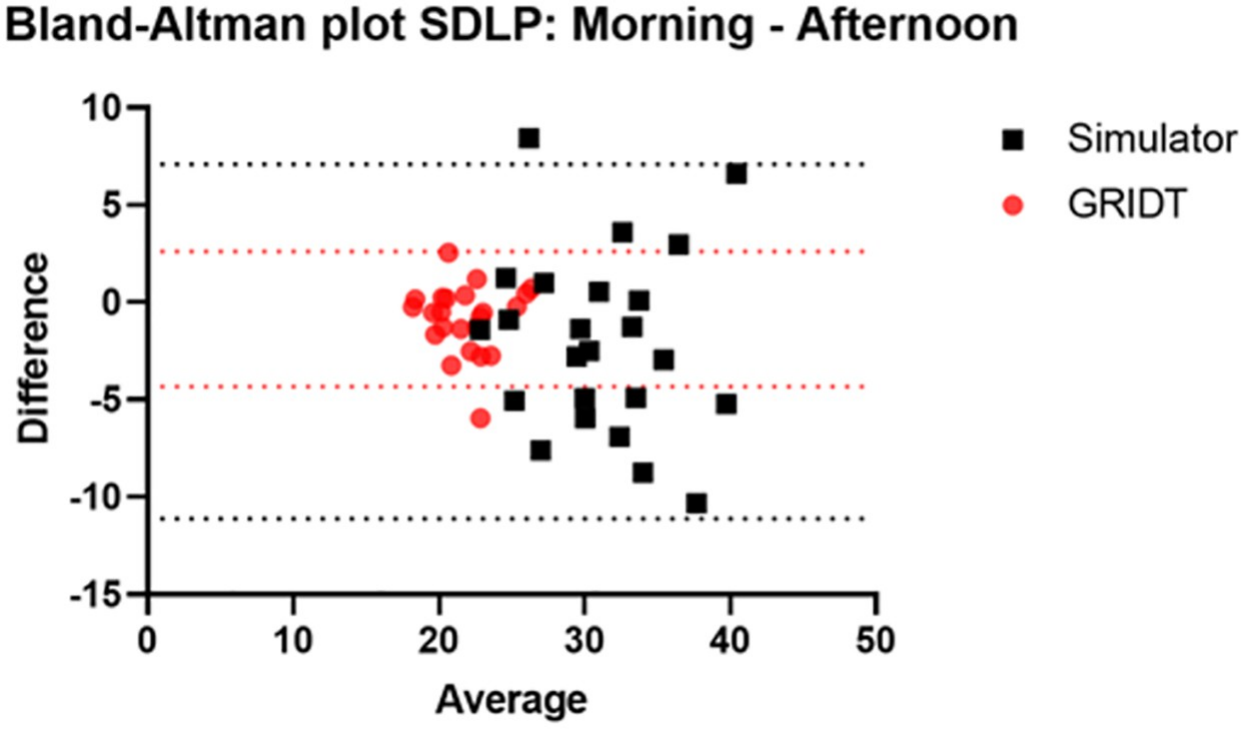
Combined Bland-Altman plot for simulator (bias: -2.01 and 95% limits of agreement: -11.10 and 7.09) and GRIDT (bias: -0.860 and 95% limits of agreement: -4.33 and 2.61).

The simulator driving parameters are presented in Table 2. The SDLP(SD) is 30.14 cm (5.2) and 32.16 cm (5.5) during the well-rested visit for morning and afternoon driving assessments, respectively. The CoV for the simulator was 11.8% and 16.5% for inter and intra subject variability, respectively (Table 1). The common variance of the SDLP measured in the simulator was 47.99 (CoV: 20.3%). The Bland-Altman plot (Fig 6) shows the bias and limits of agreement for the simulator (bias: -2.01 and 95% limits of agreement: -11.10 and 7.09) as well as for the GRIDT (bias: -0.860 and 95% limits of agreement: -4.33 and 2.61).

### Sensitivity of driving parameters

#### GRIDT

The GRIDT SDLP was significantly increased (2.76 cm, *p* < .001) after a night of sleep deprivation compared to the morning assessments after a well-rested night. While the mean speed was reduced after sleep deprivation, the SD speed was not (Table 2). Subjects report lower driving performance and motivation and increased driving effort after sleep deprivation compared to the well-rested morning. In line with the results of these self-reported questionnaires, the instructor assessment also indicated lower driving performance scores after sleep deprivation.

#### Driving simulator

The SDLP measured by the simulator was significantly increased (9.97 cm, p < .001) after a night of sleep deprivation compared to the morning assessment after a well-rested night. The SD-speed for the simulator increased significantly after a night of sleep deprivation compared to the assessments on the well-rested morning, while the mean speed was not. Like the GRIDT, subjects rate a decrement in their driving performance and motivation, and an increase in effort scores after a night of sleep deprivation compared to the well-rested morning.

### Correlation between simulator and psychomotor test battery

The mean and 95% CI results of the cognitive tests are presented in Table 3. Except for the smooth eye pursuit (p = .34), all tests performed using the NeuroCart^®^ were significantly affected by sleep deprivation. The Pearson correlations are calculated between the parameters in each condition (i.e., well-rested and sleep-deprived) and the SDLP of both the simulator and the GRIDT in that same condition (Table 4). The correlations are included in the overview when at least one of the driving assessments has a significant correlation (p < .05). For these cases the correlation with the other driving assessment (i.e., GRIDT or diving simulator) is shown, except for the test related subjective scores. The highest correlation (-0.73, p < .001) was between the driving simulator SDLP and corresponding subjective driving performance and motivation score. The smooth eye pursuit significantly correlated with the simulator SDLP (0.49 with p = .01), but not with the GRIDT SDLP (p = .41). The correlation between the driving assessment in the simulator and with the GRIDT was significant for the well-rested morning (0.63 with p < .001) and the well-rested afternoon (0.58 with p = .003). Although not presented in Table 4, the correlation was not significant for the SDLP after a night of sleep deprivation (0.31 with p = .18).

**Table 3:** Mean values (SD) of NeuroCart parameters.

Parameter	Karolinska Sleepiness Scale	Saccadic peak velocity (deg/s)	Saccadic reaction time (sec)	Smooth pursuit (%)	Body sway (mm)^1^	Adaptive tracking (%)	VAS Alertness (mm)	VAS Calmness (mm)	VAS Mood (mm)
Well rested morning (SD) n = 24	3.5 (1.2)	528.8 (62.0)	0.214 (0.03)	45.23 (9.3)	211.0 (80.8)	32.7 (4.2)	53.8 (5.3)	54.3 (5.5)	54.9 (6.7)
Well rested afternoon (SD) n = 24	4.0 (1.2)	514.4 (55.6)	0.216 (0.03)	44.63 (9.9)	204.3 (97.6)	33.1 (5.2)	52.1 (6.2)	56.5 (6.1)	55.3 (5.8)
Sleep deprived (SD) n = 23	6.8 (1.2)	489.1 (61.7)	0.222 (0.03)	44.56 (12.2)	249.0 (130.1)	26.8 (5.9)	34.7 (9.2)	61.5 (11.0)	49.7 (7.2)
Contrasts (95% CI) of Well rested morning vs Sleep deprived	3.3 (2.7, 4.0)	-40.71 (-53.25, -28.16)	0.0084 (0.0007, 0.0162)	-1.04 (-3.20, 1.13)	17.6% (2.3%, 35.1%)	-5.847 (-7.581,-4.113)	-19.18 (-23.07, -15.29)	7.23 (3.57, 10.89)	-5.27 (-7.96, -2.58)
p-value of Well rested morning vs Sleep deprived	p< .001	p< .001	p< 0.05	p = 0.34	p< 0.05	p< .001	p< .001	p< .001	p< .001

^1^ Geometric mean based on logarithmic transformed data

**Table 4:** Pearson correlations for the variables in different conditions. Only correlations with at least one significant correlation (p < .05) are included in this overview.

Variable	SDLP	Condition	(Pearson) correlation	p-value	Intercept	Slope
SDLP GRIDT	Simulator	Well rested morning	0.63	< .001	12.79	0.28
		Well rested afternoon	0.58	.003	13.68	0.26
Mean Speed simulator	Simulator	Well rested afternoon	0.21	.33	94.60	0.07
Mean Speed GRIDT	GRIDT	Sleep deprived morning	-0.54	.01	103.18	-0.34
Subj driving performance and motivation simulator	Simulator	Sleep deprived morning	-0.73	< .001	12.57	-0.20
		Well rested afternoon	-0.40	.05	13.00	-0.16
Subj driving effort simulator	Simulator	Sleep deprived morning	0.56	.01	2.05	0.20
		Well rested afternoon	0.49	.02	-3.53	0.25
Subj driving effort GRIDT	GRIDT	Sleep deprived morning	0.44	.05	-7.54	0.70
Smooth eye pursuit	Simulator	Well rested morning	0.49	.01	18.41	0.89
	GRIDT	Well rested morning	0.18	.41	30.10	0.71

**Fig 7:**
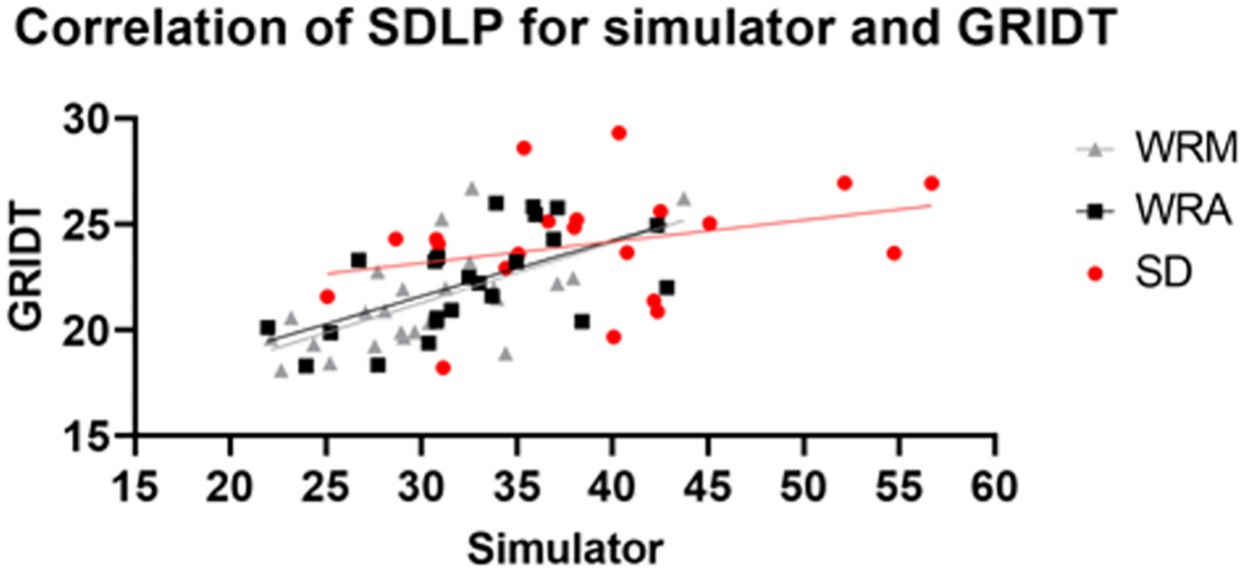
Scatter plot of the SDLP measured by the simulator and the GRIDT for the well-rested morning (WRM), well-rested afternoon (WRA) and the sleep deprived (SD) assessments.

The scatter plot in Fig 7 shows a linear correlation between the SDLP measured by the GRIDT and the simulator for each set of assessments. The correlation between both well-rested assessments was rather similar, the trendline flattens for the sleep deprived measurements.

Additional Pearson correlations were calculated for the difference in the well-rested morning measurement, and the sleep deprived measurement (see Table 5). Like Table 4, only significant correlations were shown. Four correlations were ±0.50 or stronger, but most correlations were not significant (p > .05). The adaptive tracker, which is the only NeuroCart^®^ test included in the table, had a significant correlation with the simulator of -0.50 with p = .02, but not with the GRIDT (correlation of -0.13 with p = .58).

**Table 5:** Pearson correlations for the delta between sleep deprivation and well-rested morning for any parameter and the SDLP of the simulator and GRIDT. Only correlations with at least one significant value (i.e., p ≤ .05) are presented.

Parameter	SDLP	(Pearson) correlation	P-value	Intercept	Slope
SDLP GRIDT	Simulator	0.51	.02	1.60	0.15
Subj driving performance and motivation simulator	Simulator	-0.50	.01	-2.03	-0.18
Subj driving effort GRIDT	GRIDT	0.45	.04	3.31	0.97
Adaptive Tracker	Simulator	-0.50	.02	-3.72	-0.22
	GRIDT	-0.13	.58	-4.81	-0.24
VAS Alertness	Simulator	-0.44	.04	-14.25	-0.51
	GRIDT	0.48	.03	-24.45	21.60
VAS Calmness	Simulator	-0.10	.64	8.28	-0.11
	GRIDT	-0.44	.05	13.66	-22.25
VAS Mood	Simulator	-0.53	.01	-1.32	-0.41
	GRIDT	-0.02	.95	-4.81	-0.06

## Discussion

To obtain a more detailed overview of the effect of healthcare interventions on driving performance, it is important to assess driving performance and assess both cognitive and motor performance. Here, we present the results of a study where the driving performance (both on the road and in a simulator) and the performance on a variety of psychomotor tasks were affected by sleep deprivation. Additionally, we compared the correlations between the different tasks.

### Repeatability of on-the-road and simulated driving

Instead of a camera mounted on the left backside on the car’s roof as often used in on-the-road driving test [Verster & Roth], we installed a camera system (Mobileye) behind the front window to capture the SDLP during the on-the-road task. Using this method, we observe slightly higher SDLP values under well-rested conditions than reported in other studies [16]. This difference might be explained by the different position of the camera systems and differences in the processing of raw video to the estimated lateral position. However, we observe similar SDLP values and variability in the SDLP values compared to another clinical trial using the Mobileye [37]. Additionally, the Mobileye was successfully used in other driving studies [38, 39]. Therefore, we conclude that the on-the-road values presented here are reliable.

The SDLP, mean speed, and the SD speed were repeatable during on-the-road and simulated driving (see Table 1). While the repeatability of the mean speed was similar for both simulated and on-the-road driving, the repeatability of the SDLP and SD speed were better during on-the-road driving than during simulated driving. An explanation could be that environmental conditions were constrained in the simulator (e.g., road conditions, other cars) and identical for all sessions. These constraints were not present during the on-the-road task, in which participants’ constant vigilance is required to anticipate unpredictable events. This could indicate that the driving simulator has a faster habituation effect. As suggested by Helland et al., subjects have a lower sense of danger and gravitational cues when driving in a driving simulator which are normally used to adjust steering [40]. This might explain the higher SDLP values (Table 2) and the higher variability of the SDLP of the driving simulator (Table 1). Nonetheless, we conclude that on-the-road driving performance can be assessed with better repeatability than simulated driving.

### Effect of sleep deprivation

Sleep deprivation affected all CNS task outcomes, except for the smooth eye movements, mean speed in the driving simulator, and the SD of the speed in the on-the-road car (Tables 2 and 3). This is in line with what was reported previously by others on simulated driving [41], on-the-road driving [16], and on the psychomotor test battery [29]. The psychomotor test results reported here are similar to those reported for medication-induced drowsiness [42, 43]. The on-the-road task showed a smaller effect of sleep deprivation on the SDLP than the driving simulator (13% vs 33%). However, the minimal detectable effect size (MDES) of the SDLP in the on-the-road task is lower compared to the driving simulator (1.6 vs 6.0), which can be explained by the lower variability of the SDLP in the on-the-road driving task than in the driving simulator task (Table 2). The difference in sensitivity to the effects of sleep deprivation on simulator compared to on-the-road driving indicates that the tasks are not fully interchangeable and may assess different aspects of driving behaviour.

The simulator and on-the-road SDLP values were significantly correlated during both the well-rested morning and afternoon sessions (Table 4 and Fig 7). The slope value of the linear regression lines for the well-rested morning and afternoon sessions was gradual for both sessions. Interestingly, the simulator and on-the-road SDLP values were no longer correlated during the sleep deprivation condition, while the correlation between the effect of sleep deprivation on the simulator and on-the-road SDLP values was significant (Table 5). Combined with the earlier discussed MDES, this could indicate that the simulator task is more sensitive to the effect of sleep deprivation or, that the driving tasks do not measure the same change in driving behaviour. Another explanation could be a test order effect because of the fixed order of tests in the study design. Even though both the simulator and the on-the-road task can be used to detect sleep deprivation induced changes, the lack of (strong) correlations between both tasks during sleep deprivation could indicate that simulated driving and on-the-road driving are affected by sleep deprivation differently.

### Correlations between driving and psychomotor tasks

This study aimed to compare the effect of sleep deprivation on the driving tasks and the psychomotor test battery. High correlations between the tasks indicate a higher level of validity for the psychomotor test battery. Additionally, these correlations provide information on how sleep deprivation impairs driving performance. When assessing each set of measurements separately, none of the tasks were significantly correlated with the SDLP of the driving simulator, except for the smooth eye pursuit task at well-rested morning (Table 4). Interestingly, the smooth eye pursuit is the only task that was unable to detect the effect of sleep deprivation. The absence of a significant correlation between postural balance and the simulated driving SDLP confirms the findings of Jongen et al. in 2015 [16]. In a study by Huizinga et al. assessing the effect of alcohol and alprazolam using the same driving simulator, tracker task, and eye movement tasks, there was a significant correlation using linear regression between SDLP and the psychomotor tasks. The lack of (strong) correlations between the psychomotor and driving tasks in this study indicates that care must be taken when relating psychomotor performance to driving ability.

For the effect of sleep deprivation, only a few of the correlations between the driving simulator, on-the-road driving, and psychomotor tasks were significant. Of the psychomotor tasks, the adaptive tracker showed a significant correlation with the driving simulator, but not for the on-the-road driving, for the effect of sleep deprivation (Table 5). Park et al. [44] suggested that the driving simulator task might measure different effects because of the long monotonous task compared to the short psychomotor tasks. Another reason for the insignificant correlation between the psychomotor and on-the-road tasks is that the on-the-road steering wheel and other technical properties of the car induced more heavy steering and a default position to steer straight ahead, which might help maintain a straight course, thereby effectively reducing the SDLP. These technical properties are not present in the simulator and tracker. Another explanation could be a possibly heightened sense of danger during the on-the-road assessment due to the inability of a serious crash in the simulator. This cannot be confirmed since no assessments of fear, stress or stress hormone levels were performed during the day. Any of those measurements should be included when assessing possible test-effects. The difference between the assessments is the possible anxiety for a crash and the external visual/audiological stimuli during the on-the-road driving task (such as weather and special vehicles). Even though no radio or conversations were allowed during on-the-road driving, the surroundings were less repetitive and stable than the psychomotor test battery and driving simulator. All other significant correlations for the sleep deprivation effect and the driving simulator are found with the subjective assessments of driving (performance and effort) and the VAS-BL (Table 5). However, it should be noted that the participants in this study were not blinded, which might have influenced this study’s subjective measures.

The test duration for both driving tasks was around 30 minutes. This made it possible to compare both tasks, but it does deviate from the standard length of the on-the-road tasks, which is 100 km [15]. Increasing the length of the trajectory, and therefore the duration of the task, might show a more prominent effect of sleep deprivation on the SDLP. The data for this study has not been analysed in the same way as done by Verster et al. [15]. A different cut-off for speed is used and removing of lane switches. The absolute increase of the SDLP found in this study must therefore be compared to other on-the-road driving studies with care.

### Limitations

Several limitations should be noted when interpreting the results of this study. First, the study design did not include a habituation night for both the well-rested and sleep deprivation visits. Lifestyle restrictions demanded that each participant have a stable sleep pattern with a bedtime between 22:00 and 23:00 hours and awakening between 7:00 and 8:00 hours. Although all participants confirmed adherence at the start of each visit, it cannot be ruled out that a few participants had not followed up on these restraints. While the effect of sleep deprivation was detected for most measures, the result of the study could have been optimised by adding a habituation night [45].

Secondly, differences in the interval between the two study visits between subjects may have influenced study outcomes. A 5-day interval was applied between the sleep deprivation visit and the subsequent well-rested visit for one group of participants. In contrast, the other group performed the well-rested and sleep-deprived visits contiguously. This may have led to a difference in familiarisation or learning effect between the two groups. If we had introduced a 5-day interval between the well-rested and the sleep deprivation periods, this potential bias could have been prevented. However, all subjects were trained during the screening period on all study procedures. Therefore, the learning and familiarisation effect during the actual study periods is expected to be small.

This study was performed in young, healthy men who were considered experienced drivers. This population was chosen for pragmatic reasons as a second part of the study included determining the effects of sleep deprivation on evoked pain tests, which had to be initially restricted to a male population [46, 47]. Results may have been different if elderly or female drivers had also been included [14, 48]. Additionally, less experienced drivers can overestimate their driving performance [49, 50] which might influence the results of the driving tasks and the correlations between driving variables and the psychomotor test battery. In conclusion, the selection of the study populations may limit the generalisability of the study results.

The current selection of psychomotor tasks does not cover all cognitive domains which might be influenced by sleep deprivation. Other tests often used in sleep deprivation studies, such as the PVT-192 [51], might have better correlations between the isolated testing of psychomotor functioning and the SDLP measured with either the simulator or the on-the-road car.

## Conclusion

In general, this study demonstrates that the psychomotor test battery, driving simulator and on-the-road driving tasks, are sensitive to sleep deprivation. However, the lack of apparent correlations between test variables under well-rested and sleep-deprived conditions indicates that each task in this study measures driving impairment differently. This study supports the need for studies in early drug development, including driving tasks as close to real-life driving as possible and indicates the complexity of (impaired) driving behaviour.

## Supporting information

S1 File Study protocol. (PDF)

S1 Checklist CONSORT 2010 checklist of information to include when reporting a randomised trial*. (DOC)

S1 Data (CSV)

## References

[1] Higgins J. S. et al., “Asleep at the Wheel-The Road to Addressing Drowsy Driving,” *Sleep*, vol. 40, no. 2, 2017, doi: 10.1093/sleep/zsx001 28364516

[2] CBS, “Centraal Bureau voor de Statistiek—overledenen: doden door verkeersongeval in Nederland.,” 2017.

[3] SWOV, “SWOV factsheet rijden onder invloed.”

[4] A. Foundation, “Prevalence drowsy driving crashes estimates large scale naturalistic driving study.”

[5] Bioulac S. et al., “Risk of motor vehicle accidents related to sleepiness at the wheel: A systematic review and meta-analysis,” *Sleep*, vol. 40, no. 10, 2017, doi: 10.1093/sleep/zsx134 28958002

[6] FDA—CDER, “Evaluating Drug Effects on the Ability to Operate a Motor Vehicle—Guidance for Industry,” 2017. [Online]. https://www.fda.gov/Drugs/GuidanceComplianceRegulatoryInformation/Guidances/default.htm

[7] Helland A. et al., “Comparison of driving simulator performance with real driving after alcohol intake: A randomised, single blind, placebo-controlled, cross-over trial,” *Accid Anal Prev*, vol. 53, pp. 9–16, 2013, doi: 10.1016/j.aap.2012.12.042 23357031

[8] Veldstra J. L., Bosker W. M., de Waard D., Ramaekers J. G., and Brookhuis K. A., “Comparing treatment effects of oral THC on simulated and on-the-road driving performance: Testing the validity of driving simulator drug research,” *Psychopharmacology (Berl)*, vol. 232, no. 16, pp. 2911–2919, Aug. 2015, doi: 10.1007/s00213-015-3927-9 25957748 PMC4513227

[9] Huizinga C. R. et al., “Evaluation of simulated driving in comparison to laboratory-based tests to assess the pharmacodynamics of alprazolam and alcohol,” *J Psychopharmacol*, p. 269881119836198, 2019, doi: 10.1177/0269881119836198 30912701

[10] Simen A. A. et al., “A randomized, crossover, placebo-controlled clinical trial to assess the sensitivity of the CRCDS Mini-Sim to the next-day residual effects of zopiclone,” *Ther Adv Drug Saf*, vol. 6, no. 3, pp. 86–97, 2015, doi: 10.1177/2042098615579314 26240742 PMC4519739

[11] Mets M. A. J., Kuipers E., de Senerpont Domis L. M., Leenders M., Olivier B., and Verster J. C., “Effects of alcohol on highway driving in the STISIM driving simulator,” *Hum Psychopharmacol*, vol. 26, no. 6, pp. 434–439, Aug. 2011, doi: 10.1002/hup.1226 21823173

[12] Verster J. C., Taillard J., Sagaspe P., Olivier B., and Philip P., “Prolonged nocturnal driving can be as dangerous as severe alcohol-impaired driving,” *J Sleep Res*, vol. 20, no. 4, pp. 585–588, Dec. 2011, doi: 10.1111/j.1365-2869.2010.00901.x 21226780

[13] J. C. Verster, D. W. Spence, A. Shahid, S. R. Pandi-Perumal, and T. Roth, “Zopiclone as Positive Control in Studies Examining the Residual Effects of Hypnotic Drugs on Driving Ability,” 2011. 10.2174/157488611798280933 22129315

[14] Jongen S. et al., “A pooled analysis of on-the-road highway driving studies in actual traffic measuring standard deviation of lateral position (i.e., ‘weaving’) while driving at a blood alcohol concentration of 0.5 g/L,” *Psychopharmacology (Berl)*, vol. 234, no. 5, pp. 837–844, Mar. 2017, doi: 10.1007/s00213-016-4519-z 28070617 PMC5306436

[15] Verster J. and Roth T., “Standard operation procedures for conducting the on-the-road driving test, and measurement of the standard deviation of lateral position (SDLP),” *Int J Gen Med*, p. 359, 2011, doi: 10.2147/IJGM.S19639 21625472 PMC3100218

[16] Jongen S., Perrier J., Vuurman E. F., Ramaekers J. G., and Vermeeren A., “Sensitivity and validity of psychometric tests for assessing driving impairment: Effects of sleep deprivation,” *PLoS One*, vol. 10, no. 2, Feb. 2015, doi: 10.1371/journal.pone.0117045 25668292 PMC4323110

[17] Verster J. and Roth T., “Standard operation procedures for conducting the on-the-road driving test, and measurement of the standard deviation of lateral position (SDLP),” *Int J Gen Med*, p. 359, 2011, doi: 10.2147/IJGM.S19639 21625472 PMC3100218

[18] Jacobs M., Hart E. P., Miranda Y. M., Groeneveld G. J., van Gerven J. M. A., and Roos R. A. C., “Altered driving performance of symptomatic Huntington’s disease gene carriers in simulated road conditions,” *Traffic Inj Prev*, vol. 19, no. 7, pp. 708–714, 2018. doi: 10.1080/15389588.2018.1497796 30273496

[19] Huizinga C. R. et al., “Evaluation of simulated driving in comparison to laboratory-based tests to assess the pharmacodynamics of alprazolam and alcohol,” *J Psychopharmacol*, p. 269881119836198, 2019, doi: 10.1177/0269881119836198 30912701

[20] AL. van Steveninck, “Methods of assessment of central nervous system effects of drugs in man.,” 1993.

[21] Van Steveninck A. L., Schoemaker H. C., Pieters M. S. M., Kroon R., Breimer D. D., and Cohen A. F., “A comparison of the sensitivities of adaptive tracking, eye movement analysis, and visual analog lines to the effects of incremental doses of temazepam in healthy volunteers,” *Clin Pharmacol Ther*, vol. 50, no. 2, pp. 172–180, 1991, doi: 10.1038/clpt.1991.122 1868679

[22] Baloh R., Sills A., Kumley W., and Honrubia V., “Quantitative measure¬ment of saccade amplitude, duration and velocity.,” *Neurology*, vol. 25, pp. 1065–1070, 1975. 1237825 10.1212/wnl.25.11.1065

[23] Van Steveninck A., Cohen A., and Ward T., “A microcomputer based system for recording and analysis of smooth pursuit and saccadic eye movements.,” *Brit*.*J*.*Clin*.*Pharmaco*., vol. 27, no. 5, pp. 712–713, 1989.

[24] AL. Van Steveninck, “Methods of assessment of central nervous system effects of drugs in man.,” State University Leiden, 1993.

[25] Bittencourt P., Wade P., Smith A., and Richens A., “Benzodiazepines impair smooth pursuit eye movements.,” *Br J Clin Pharmacol*, 1983, doi: 10.1111/j.1365-2125.1983.tb01495.x 6133544 PMC1427870

[26] Baloh R., Sills A., Kumley W., and Honrubia V., “Quantitative measurement of saccade amplitude, duration and velocity.,” *Neurology*, vol. 25, pp. 1065–1070, 1975. 1237825 10.1212/wnl.25.11.1065

[27] van Steveninck A. L. et al., “Pharmacodynamic interactions of diazepam and intravenous alcohol at pseudo steady state,” *Psychopharmacology (Berl)*, 1993, doi: 10.1007/BF02244655 7870919

[28] Groeneveld G. J., Hay J. L., and Van Gerven J. M., “Measuring blood–brain barrier penetration using the NeuroCart, a CNS test battery,” *Drug Discovery Today*: *Technologies*, vol. 20. Elsevier Ltd, pp. 27–34, Jun. 01, 2016. doi: 10.1016/j.ddtec.2016.07.004 27986220

[29] van Steveninck A. L., van Berckel B. N. M., Schoemaker R. C., Breimer D. D., van Gerven J. M. A., and Cohen A. F., “The sensitivity of pharmacodynamic tests for the central nervous system effects of drugs on the effects of sleep deprivation,” *Journal of Psychopharmacology*, vol. 13, no. 1, pp. 10–17, 1999, doi: 10.1177/026988119901300102 10221355

[30] Wright BM., “A simple mechanical ataxia-meter.,” *J Physiol*, vol. 218, pp. 27P–28P, 1971. 5130616

[31] Van Steveninck A. L., Van Berckel B. N. M., Schoemaker R. C., Breimer D. D., Van Gerven J. M. A., and Cohen A. F., “The sensitivity of pharmacodynamic tests for the central nervous system effects of drugs on the effects of sleep deprivation,” *Journal of Psychopharmacology*, 1999, doi: 10.1177/026988119901300102 10221355

[32] van Steveninck A. L. et al., “Pharmacodynamic interactions of diazepam and intravenous alcohol at pseudo steady state,” *Psychopharmacology (Berl)*, vol. 110, no. 4, pp. 471–478, 1993, doi: 10.1007/BF02244655 7870919

[33] Norris H., “The action of sedatives on brain stem oculomotor systems in man,” *Neuropharmacology*, 1971, doi: 10.1016/0028-3908(71)90039-6 5093959

[34] de Visser S. J., van der Post J., Pieters M. S. M., Cohen A. F., and van Gerven J. M. A., “Biomarkers for the effects of antipsychotic drugs in healthy volunteers,” *Br J Clin Pharmacol*, 2001, doi: 10.1111/j.1365-2125.2001.01308.x 11259983 PMC2014436

[35] Bond M., Lader A., “The use of analogue scales in rating subjective feelings,” *Br J Med Psychol*, vol. 47, pp. 211–218, 1974.

[36] Åkerstedt T. and Gillberg M., “Subjective and objective sleepiness in the active individual,” *International Journal of Neuroscience*, vol. 52, no. 1–2, pp. 29–37, 1990, doi: 10.3109/00207459008994241 2265922

[37] Anund A., Fors C., Hallvig D., Åkerstedt T., and Kecklund G., “Observer Rated Sleepiness and Real Road Driving: An Explorative Study,” *PLoS One*, vol. 8, no. 5, May 2013, doi: 10.1371/journal.pone.0064782 23724094 PMC3665781

[38] Sun Y., Wu C., Zhang H., Zhang Y., Li S., and Feng H., “Extraction of Optimal Measurements for Drowsy Driving Detection considering Driver Fingerprinting Differences,” *J Adv Transp*, vol. 2021, 2021, doi: 10.1155/2021/5546127

[39] Zhang H., Wu C., Huang Z., Yan X., and Qiu T. Z., “Sensitivity of lane position and steering angle measurements to driver fatigue,” *Transp Res Rec*, vol. 2585, pp. 67–76, 2016, doi: 10.3141/2585-08

[40] Helland A. et al., “Comparison of driving simulator performance with real driving after alcohol intake: A randomised, single blind, placebo-controlled, cross-over trial,” *Accid Anal Prev*, vol. 53, pp. 9–16, 2013, doi: 10.1016/j.aap.2012.12.042 23357031

[41] Garner A. A., Miller M. M., Field J., Noe O., Smith Z., and Beebe D. W., “Impact of experimentally manipulated sleep on adolescent simulated driving,” *Sleep Med*, vol. 16, no. 6, pp. 796–799, Jun. 2015, doi: 10.1016/j.sleep.2015.03.003 25953298 PMC4449807

[42] Schrier L. et al., “Pharmacokinetics and pharmacodynamics of a new highly concentrated intranasal midazolam formulation for conscious sedation,” *Br J Clin Pharmacol*, vol. 83, no. 4, pp. 721–731, 2017, doi: 10.1111/bcp.13163 27780297 PMC5346864

[43] Groeneveld G. J., Hay J. L., and van Gerven J. M., “Measuring blood–brain barrier penetration using the NeuroCart, a CNS test battery,” *Drug Discovery Today*: *Technologies*, vol. 20. Elsevier Ltd, pp. 27–34, Jun. 01, 2016. doi: 10.1016/j.ddtec.2016.07.004 27986220

[44] D. Park, J. C. Ware, J. F. May, T. J. Rosenthal, M. R. Guibert, and R. W. Allen, “The Effects of Sleep Deprivation on Simulator Driving as Compared with Other Psychomotor Tests George,” 2007, pp. 257–264.

[45] P. Alhola and P. Polo-Kantola, “Sleep deprivation: Impact on cognitive performance,” 2007. PMC2656292 19300585

[46] H. J. Hijma, I. W. Koopmans, E. Klaassen, R. J. Doll, R. G. J. A. Zuiker, and G. J. Groeneveld, “A randomized, crossover study to evaluate the gender and sensitizing effects of sleep deprivation using a nociceptive test battery in healthy volunteers,” *submitted*. 10.1111/bcp.15505 PMC10086808 35997713

[47] van den Berg B. et al., “Simultaneous measurement of intra-epidermal electric detection thresholds and evoked potentials for observation of nociceptive processing following sleep deprivation,” *Exp Brain Res*, Jan. 2022, doi: 10.1007/s00221-021-06284-5 34993590 PMC8739349

[48] Michaels J. et al., “Driving simulator scenarios and measures to faithfully evaluate risky driving behavior: A comparative study of different driver age groups,” *PLoS One*, vol. 12, no. 10, Oct. 2017, doi: 10.1371/journal.pone.0185909 29016693 PMC5634611

[49] de Craen S., Twisk D. A. M., Hagenzieker M. P., Elffers H., and Brookhuis K. A., “Do young novice drivers overestimate their driving skills more than experienced drivers? Different methods lead to different conclusions,” *Accid Anal Prev*, vol. 43, no. 5, pp. 1660–1665, Sep. 2011, doi: 10.1016/j.aap.2011.03.024 21658492

[50] Rosenbloom T., Beigel A., Perlman A., and Eldror E., “Parental and offspring assessment of driving capability under the influence of drugs or alcohol: Gender and inter-generational differences,” *Accid Anal Prev*, vol. 42, no. 6, pp. 2125–2131, 2010, doi: 10.1016/j.aap.2010.07.002 20728671

[51] Lim J. and Dinges D. F., “Sleep deprivation and vigilant attention,” in *Annals of the New York Academy of Sciences*, 2008, vol. 1129, pp. 305–322. doi: 10.1196/annals.1417.002 18591490

