# Supplementary material for: Fit for purpose of on-the-road driving and simulated driving: A randomised crossover study using the effect of sleep deprivation
Source: PLoS One. 2023 Feb 2;18(2):e0278300. doi: 10.1371/journal.pone.0278300 (PMC9894419; doi:10.1371/journal.pone.0278300)
Supplement: S1 File — (PDF) [file pone.0278300.s001.pdf]

CLINICAL STUDY PROTOCOL

An exploratory single-centre cross-over study in healthy subjects to investigate the effects of sleep deprivation on driving, EEG, and PainCart.

Short Title:	Effects of sleep deprivation on driving, EEG, and PainCart.
Version:	3
Date:	08-Mar-2019
CHDR number:	CHDR1818
Toetsing Online number:	NL68626.056.19

CONTACT DETAILS

**Trial Site**<br>**(visit & delivery address)**	Centre for Human Drug Research<br>Zernikedreef 8<br>2333 CL Leiden<br>The Netherlands<br>Telephone: + 31 71 5246 400<br>Fax: + 31 71 5246 499<br>Emergency: + 31 71 5246 444
Principal investigator	R.G.J.A. Zuiker, MD, PhD<br>Telephone: + 31 71 5246 400<br>e-mail: rzuiker@chdr.nl
Co-investigator	G.J. Groeneveld, MD, PhD<br>Telephone: + 31 71 5246 400<br>e-mail: ggroeneveld@chdr.nl
Co-investigator	A.F. Cohen, MD, PhD<br>Telephone: + 31 71 5246 400<br>e-mail: ac@chdr.nl
Co-investigator	R.J. Doll, PhD<br>Telephone: + 31 71 7517 162<br>e-mail: rjdoll@chdr.nl
Co-investigator	I.W. Koopmans, MSc<br>Telephone: + 31 71 7517 189<br>e-mail: ikoopmans@chdr.nl
Co-investigator	H.J. Hijma, MSc<br>Telephone: + 31 71 7517 185<br>e-mail: hhijma@chdr.nl
Co-investigator	H.E.C. van der Wall, MSc<br>Telephone: + 31 71 5246 400<br>e-mail: hvdwall@chdr.nl
Manager Operations Unit	J. M. (Ria) Kroon, BSc<br>Telephone: + 31 71 5246 498<br>e-mail: rk@chdr.nl
Manager Clinical Unit	C. E. (Emilie) Jonxis, MANP<br>Telephone +31 71 5246 433<br>e-mail: ejonxis@chdr.nl
Statistician	M. L. (Marieke) de Kam, MSc<br>Telephone: + 31 71 5246 458<br>e-mail: mdekam@chdr.nl
**INDEPENDENT PHYSICIAN**	Prof. Dr G.J. Blauw, MD, PhD<br>Department of Gerontology and Geriatrics LUMC<br>Postbus 9600<br>2300 RC Leiden<br>Telephone: + 31 71 5266 640

CHDR template v2018.9

SIGNATURE PAGE - PRINCIPAL INVESTIGATOR

Study Title

An exploratory single-centre cross-over study in healthy subjects to investigate the effects of sleep deprivation on driving, EEG, and PainCart.

I acknowledge accountability for this protocol in accordance with CHDR's current procedures.

R.G.J.A. Zuiker, MD, PhD<br>Principal investigator	Signature	11 Mrt 2019<br>Date (dd Mmm yyyy)

SIGNATURE PAGE - TRIAL SITE STAFF
Centre for Human Drug Research

Study Title

An exploratory single-centre cross-over study in healthy subjects to investigate the effects of sleep deprivation on driving, EEG, and PainCart.

I acknowledge responsibility for this protocol in accordance with CHDR's current procedures.

Name / Role	Signature	Date (dd Mmm yyyy)
G.J. Groeneveld, MD, PhD<br>Co-investigator	[signature]	11 MAR 2019
A.F. Cohen, MD, PhD<br>Co-investigator	[signature]	11 MAR 2019
R.J. Doll, PhD<br>Co-investigator	[signature]	11 MAR 2019
I.W. Koopmans, MSc<br>Co-investigator	[signature]	11-Mar-2019
H.J. Hijma, MSc<br>Co-investigator	[signature]	11-Mar 2019
H. van der Wall, MSc<br>Co-investigator	[signature]	11 MAR 2019
J. M. (Ria) Kroon, BSc<br>Manager Operations Unit	[signature]	11Mar2019.
C.E. (Emilie) Jonxis, MANP<br>Manager Clinical Unit	[signature]	11 MAR 2019
M. L. (Marieke) de Kam, MSc<br>Statistician	* [signature]	11 MAR 2019

*E Klaassen
Statistician

TABLE OF CONTENTS

CONTACT DETAILS 2

SIGNATURE PAGE - PRINCIPAL INVESTIGATOR 3

SIGNATURE PAGE - TRIAL SITE STAFF 4

TABLE OF CONTENTS 5

LIST OF ABBREVIATIONS 8

1 BACKGROUND AND RATIONALE 10

1.1 Context 10

1.2 Study rationale 11

1.2.1 Benefit and risk assessment 11

1.2.2 Study population 11

1.2.3 Study design 11

1.2.4 Safety margin calculations, dose selection, dose escalation, and stopping criteria 11

1.2.5 Treatment duration 11

1.2.6 Endpoints 11

1.2.7 Statistical hypotheses and sample size 12

2 STUDY OBJECTIVES 13

3 STUDY DESIGN 16

3.1 Overall study design and plan 16

3.1.1 Screening 17

3.1.2 Intervention and observation period 17

4 STUDY POPULATION 18

4.1 Subject population 18

4.2 Inclusion criteria 18

4.3 Exclusion criteria 18

4.4 Concomitant medications 18

4.5 Lifestyle restrictions 18

4.6 Study drug discontinuation and withdrawal 19

4.6.1 Study drug interruption or discontinuation 19

4.6.2 Subject withdrawal 19

4.6.3 Replacement policy 19

5 INVESTIGATIONAL MEDICINAL PRODUCT 20

6 STUDY ENDPOINTS 21

6.1 Safety and tolerability endpoints 21

6.2 Pharmacodynamic endpoints 21

7 STUDY ASSESSMENTS 23

7.1 Efficacy assessments 23

7.2 Safety and tolerability assessments 29

7.2.1 Investigator assessment during driving test 29

8 SAFETY REPORTING 30

8.1 Definitions of adverse events 30

8.1.1 Intensity of adverse events 30

8.1.2 Chronicity of adverse events 30

8.1.3 Action 30

8.1.4 Serious adverse events 30

8.1.5 Reporting of serious adverse events 30

8.1.6 Follow-up of adverse events 31

8.2 Temporary halt for reasons of subject safety 31

9 STATISTICAL METHODOLOGY AND ANALYSES 32

9.1 Statistical analysis plan 32

9.2 Protocol violations/deviations 32

9.3 Power calculation 32

9.4 Missing, unused and spurious data 32

9.5 Analysis sets 32

9.5.1 Safety set 32

9.5.2 Pharmacodynamic analysis set 32

9.6 Subject disposition 33

9.7 Baseline parameters and concomitant medications 33

9.7.1 Demographics and baseline variables 33

9.7.2 Medical history 33

9.8 Safety and tolerability endpoints 33

9.8.1 Adverse events 33

9.8.1 Prematurely discontinued on-the-road driving tests 33

9.9 Pharmacodynamics endpoints 33

9.10 Exploratory analyses and deviations 34

10 GOOD CLINICAL PRACTICE, ETHICS AND ADMINISTRATIVE PROCEDURES 35

10.1 Good clinical practice 35

10.1.1 Ethics and good clinical practice 35

10.1.2 Ethics committee / institutional review board 35

10.1.3 Informed consent 35

10.1.4 Insurance 35

10.2 Study funding 36

10.3 Data handling and record keeping 36

10.3.1 Data collection 36

10.3.2 Database management and quality control 36

10.4 Access to source data and documents 36

10.5 Quality control and quality assurance 36
10.5.1 Monitoring 36
10.6 Protocol amendments 36
10.6.1 Substantial amendment 36
10.6.2 Non-substantial amendment 36
10.6.3 Urgent amendment 37
10.7 End of study report 37
10.8 Public disclosure and publication policy 37
11 STRUCTURED RISK ANALYSIS 38
12 REFERENCES 39
APPENDIX 1. PERCEIVED DRIVING QUALITY SCALE (ENGLISH VERSION). 42
APPENDIX 2. PERCEIVED DRIVING QUALITY SCALE (DUTCH VERSION). 43
APPENDIX 3. INSTRUCTOR ASSESSMENT OF DRIVING QUALITY (ENGLISH VERSION). 44
APPENDIX 4. INSTRUCTOR ASSESSMENT OF DRIVING QUALITY (DUTCH). 45
APPENDIX 5. KAROLINSKA SLEEPINESS SCALE (ENGLISH VERSION). 46
APPENDIX 6. KAROLINSKA SLEEPINESS SCALE (DUTCH VERSION). 46
APPENDIX 7. LEEDS SLEEP EVALUATION QUESTIONNAIRE (ENGLISH VERSION). 47
APPENDIX 8. LEEDS SLEEP EVALUATION QUESTIONNAIRE (DUTCH VERSION). 48
APPENDIX 9. VISUAL ANALOGUE SCALE BOND-LADER (ENGLISH VERSION). 49
APPENDIX 10. VISUAL ANALOGUE SCALE BOND-LADER (DUTCH VERSION). 50
APPENDIX 11. MCGILL PAIN QUESTIONNAIRE SHORT VERSION (SF-MPQ) (ENGLISH VERSION). 51
APPENDIX 12. MCGILL PAIN QUESTIONNAIRE SHORT VERSION (SF-MPQ) (DUTCH VERSION). 52

LIST OF ABBREVIATIONS

AAC	Area Above the Curve
AE	Adverse Event
ABR	ABR form, General Assessment and Registration form, is the application form that is required for submission to the accredited Ethics Committee; in Dutch, ABR = Algemene Beoordeling en Registratie
AUC	Area Under the Curve
BMI	Body Mass Index
BP	Blood Pressure
BrAlc	Breath Alcohol test
CA	Competent authority (also CCMO)
CHDR	Centre for Human Drug Research
CNS	Central Nerve System
CRF	Case Report Form
CRU	Clinical Research Unit
CV	Coefficient of Variation
EC	Ethics Committee (also Medical Research Ethics Committee (MREC); in Dutch: Medisch Ethische Toetsing Commissie (METC).
EEG	Electroencephalography
ERP	Evoked Response Potentials
EU	European Union
FDA	Food and Drug Administration
GCP	Good Clinical Practice
HR	Heart Rate
ICH	International Conference on Harmonization
IES	Intra-epidermal stimulation
IESEP	Intra-epidermal electrical stimulation evoked potentials
KSS	Karolinska Sleepiness Scale
LEP	Laser-evoked Potentials
LS	Laser Stimulation
LSEQ	Leeds Sleep Evaluation Questionnaire
MedDRA	Medical Dictionary for Regulatory Activities
MMN	Mismatch Negativity
MS	Mean Speed
OTC	Over The Counter
PD	Pharamacodynamic
PDT	Pain Detection Threshold
PI	Principal Investigator
PT	Preferred Term
PTT	Pain Pain Tolerance Threshold
RR	Respiratory Rate
SAE	Serious Adverse Event
SAP	Statistical Analysis Plan
SCR	Screening
SD	Standard Deviation
SDLP	Standard Deviation of Lateral Position
SDS	Standard Deviation of Speed

SEM	Standard Error of the Mean
SOC	System Organ Class
SOP	Standard Operating Procedure
UrDrug	Urine Drug screening
VAS	Visual Analogue Scale
VEP	Visual-evoked Potentials
WBP	Personal Data Protection Act; in Dutch: Wet Bescherming Persoonsgevens
WMO	Medical Research Involving Human Subjects Act; in Dutch: Wet Medisch-wetenschappelijk Onderzoek met Mensen.

1 BACKGROUND AND RATIONALE

1.1 Context

The assessment of potential sedative effects of new drugs is an important part of the early clinical drug development process. Undesired sedative drug effects may have important consequences, such as an increased risk of traffic accidents. Various methods are used to quantify sedative drug effects [1, 2]. This study assesses the sensitivity of additional measures of sedation, including driving behaviour and brain responses to various sensory and cognitive tasks. The Centre for Human Drug Research (CHDR) aims to further validate these methods by demonstrating that they can detect the effects of sleep deprivation. Additionally, sleep deprivation also affects the perception of pain (e.g., lower pain thresholds). In this study the effects of sleep deprivation on a battery of pain tests will also be determined.

Driving studies have been conducted to assess the effects of fatigue, alcohol, drugs of abuse or pharmaceutical agents on driving performance [3-5]. The first driving studies were closed road tests and a combination of laboratory tests to estimate driving performance [6]. These tests have limitations and do not represent normal driving behaviour, which for example includes dynamic interactions with other drivers. An improved version, the standardised on-the-road highway test, includes about 1 hour driving on the public highway with a constant speed while measuring the lateral position of the car with a camera system [7]. Driving behaviour can be characterised with several parameters, e.g. increased swaying in the driving lane, episodes of lane crossings, and difficulty in maintaining a constant speed [7-9]. A similar task with the same end-points can be performed in a more standardised and safe environment with a driving simulator [10]. This was also noted by the FDA in a recently published guideline, considering driving studies with actual motor vehicles and driving simulators equally for registration purposes [11]. This study will research the effect of sleep deprivation on both on-the-road and simulated driving, using the Green Dino driving simulator. This simulator has previously been used in studies including alcohol, sleep medication, and Huntington patients [12, 13]. Additionally, the relationship between driving- simulation and NeuroCart®, a validated test battery that quantifies a large range of drug-sensitive CNS-functions and that has been sensitive to the effects of sleep deprivation [2] will be assessed.

In a previous sleep deprivation study performed at the CHDR, EEG parameters for drowsiness were less affected than expected [2]. However, the applied EEG method was limited to a 1-minute resting EEG (eyes closed) only and only a limited set of electrodes were included. Other studies observed changes in the power spectrum for specific sites and changes in evoked response potentials (ERP) after sleep deprivation [14-16]. With the present study we want to further explore the relationship between sleep deprivation and a wide range of EEG parameters as well as P300 and mismatch negativity (MMN). In addition to the EEG tests, we want to include simple cognitive tasks to capture the effect of sleep deprivation on the different domains of the brain.

Sleep deprivation is a risk factor for the development of chronic pain. Central sensitization – characterized by an increased sensitivity to pain (i.e. hyperalgesia) – is found in subjects with sleep disturbances [17, 18]. Similarly, multiple studies reported that sleep-deprived subjects respond differently to evoked pain tests in a controlled setting, but is still a topic of discussion as other clinical studies provided conflicting results [19]. This may be explained by the large heterogeneity of study populations and study designs, as not all studies included a wide range of nociceptive modalities [20-23]. The design of the current study provides CHDR with the opportunity to both assess the effects of sleep deprivation on its elaborate battery of evoked pain tests (PainCart® [24]), as well as to validate

the use of sleep deprivation as a tool to induce hyperalgesia. If validated, the latter may be used as a model in drug development to demonstrate effects of centrally acting analgesic compounds.

Moreover, the effect of sleep deprivation on laser-evoked potentials (LEPs) and intra-epidermal electrical stimulation (IES) will also be assessed. For assessing sleep-deprived hyperalgesia (i.e., the increased sensitivity to pain), LEPs will be recorded with EEG after a laser stimulus (LS) is given on a pre-defined area on the volar forearm. Intra-epidermal electrical stimulation (IES) is an experimental method to measure nociceptive detection thresholds using electrical stimulation. Quantification of electrophysiological nociceptive activity can be done simultaneously by using the stimuli as events in evoked potential analysis using EEG recordings. Finally, the effects of sleep deprivation on pain will be assessed psychophysically using the McGill Pain Questionnaire after each individual pain test and LEP.

This study has two goals:

1. To measure sleep deprivation effects with various methods in order to clinically validate these methods, and to correlate the results.
2. To design a pain model based on central sensitisation.

The study is split in two parts, to reduce the risk of contaminating one test's results with effects of the other. The two parts measure the effect of sleep deprivation on:

A. Cognition and driving behaviour.
B. Response to evoked potentials and pain sensitization.

1.2 Study rationale

1.2.1 Benefit and risk assessment

There is no benefit for the subjects in this study. The risk of the intervention is negligible. For the on-the-road test are safety precautions taken: a driving instructor has access to dual controls to intervene when needed for safety reasons and the subject is instructed about the possibility to stop the driving test before the planned ending.

1.2.2 Study population

The study consists of two parts (Part A and B). For both parts the population will be 24 healthy male subjects, 23 to 35 years of age. Participants can enrol for either part A, part B or both parts.

1.2.3 Study design

An exploratory single-centre cross-over study in healthy subjects. There is no blinding included because of the nature of the study design. Subjects will be randomized on the order of sleep and sleep deprivation.

1.2.4 Safety margin calculations, dose selection, dose escalation, and stopping criteria

Not Applicable.

1.2.5 Treatment duration

The intervention of sleep deprivation will be 30 hours per study period with at least 5 days rest after sleep deprivation.

1.2.6 Endpoints

As the main focus of this study is to evaluate the effects of sleep deprivation, all related parameters are considered as main endpoints.

1.2.7 Statistical hypotheses and sample size

Part A

The population size is based on the most variable method of this part, being the driving simulator.
A sample size of 24 will have a power of 0.80 to detect a difference in means of 3.88 cm, assuming a standard deviation of differences of 6.5 cm, using a paired t-test with a 0.05 two-sided significance level.

Part B

The population size is based on the most variable method of this part, being the PainCart.
For the cold pressor pain test, a sample size of 24 will have a power of 0.80 to detect a difference in means of 2.09 (sec), assuming a standard deviation of differences of 3.5 (sec), using a paired t-test with a 0.050 two-sided significance level.

2 STUDY OBJECTIVES

Part A

- To assess the effect of sleep deprivation on next morning driving (both on road and in simulated driving) and subjective self-reported driving performance tests;
- To assess the effect of sleep deprivation on CNS functioning using the NeuroCart, a CNS test battery;
- To establish the relationship between on-the-road driving, simulated driving, and NeuroCart performance;
- To estimate the repeatability of standard deviation of the lateral position (SDLP) (both on road and in simulated driving) at day time at two different time points after a regular night of sleep.

Part B

- To assess the effect of sleep deprivation on event related potentials and EEG
- To assess the effect of sleep deprivation on pain tolerance / pain detection threshold using the PainCart, a pain test battery;
- To investigate the effect of sleep deprivation on IES sensitivity and LEPs;
- To estimate the repeatability of IES and LEP at day time at two different time points after a regular night of sleep.

Table 1. Visit and Assessment Schedule – Part A

Assessment / Time point	SCR Up to -21 d	Study Part A		
		Study period 1 Well rested state[1]		Study period 2 Sleep deprived state[1]
Projected start time assessments		Start at 9:00 hr	Start at 14:00 hr	Start at 9:00 hr
Informed consent	X			
Demographics	X			
In- and exclusion criteria	X			
Physical examination	X			
Medical history	X			
Vital Signs (HR, BP,RR)	X			
Meal		X	X	X
UrDrug, BrAlc	X	X		X
Symptoms, (S)AEs, conmed	←	→	→	→
Questionnaires[2]	X[4]	X	X	X
NeuroCart assessments[3]	X[4]	X	X	X
Simulated driving	X[4]	X	X	X
On-the-road driving	X[4]	X	X	X
Instructor assessment		X	X	X
Subjective assessment	X[4]	X[5]	X[5]	X[5]
Discharge	X		X	X[6]

AE = Adverse Event, HR = Heart Rate, BP = Blood Pressure, BrAlc = Breath Alcohol Test, UrDrug = Urine Drug SCR = Screen, RR = Respiratory Rate.

1. Subjects will be randomized over de order of well rested state and sleep deprived state. After the sleep deprived state at least five days are taken as interval.
2. Questionnaire order: Karolinska Sleepiness Scale (KSS), Leeds Sleep Evaluation Questionnaire (LSEQ).
3. NeuroCart test battery tests order: eye movements, adaptive tracker, VAS Bond & Lader, body sway.
4. For training purposes only.
5. After on the road driving & simulated driving
6. Subjects are not allowed to drive home.

Table 2. Visit and Assessment Schedule – Part B

Assessment \ Time point	SCR Up to -21 d	Study Part B		
		Study period 1 Well rested state[1]		Study period 2 Sleep deprived state[1]
Projected start time assessments		Start at 9:00 hr	Start at 14:00 hr	Start at 9:00 hr
Informed consent	X			
Demography	X			
In- and exclusion criteria	X			
Physical examination	X			
Medical history	X			
Vital Signs (HR, BP,RR)	X			
Meal		X	X	X
UrDrug, BrAlc	X	X		X
Symptoms, (S)AEs, conmed	←———	———	———	———→
Questionnaires[2]	X[5]	X		X
NeuroCart assessments[3]	X[5]	X	X	X
PainCart assessments[4]	X[5]	X	X	X
Discharge	X		X	X[6]

AE = Adverse Event, HR = Heart Rate, BP = Blood Pressure, BrAlc = Breath Alcohol Test, UrDrug = Urine Drug SCR = Screen, RR = Respiratory Rate.

1. Subjects will be randomized over de order of well rested state and sleep deprived state. After the sleep deprived state at least five days are taken as interval.
2. Questionnaire order: Karolinska Sleepiness Scale (KSS), Leeds Sleep Evaluation Questionnaire (LSEQ).
3. NeuroCart test battery tests order: eye movements, adaptive tracker, VAS Bond & Lader, body sway, resting EEG, EEG tasks.
4. PainCart test battery tests order: heat pain, pressure pain, electrical burst, electrical stair (1), cold pressor, electrical stair (2) IES and LEP will follow PainCart tests.
5. For training purposes only.
6. Subjects are not allowed to drive home.

3 STUDY DESIGN

3.1 Overall study design and plan

The study will be divided into two parts (part A and B). Subjects will be screened for eligibility within 21 days prior to study period 1 in part A, after having given their written informed consent. Subjects who have completed part A may continue with study period 1 in part B, taking into account an additional interval of at least 5 days between the study parts. Subject who are new to the study and enrol for part B, will provide a written informed consent and will be screened with 21 days period to study period 1 in part B.

Part A and B have an identical design and plan. The assessments differ according to the objectives:
- Part A is a single-centre, two-period cross-over study in healthy subjects in which the effect of sleep deprivation on driving and cognitive functions will be evaluated.
- Part B is a single-centre, two period cross-over study in healthy subjects in which the effect of sleep deprivation on EEG and pain thresholds will be evaluated.

<u>Design for each part</u>
Subjects will be randomized over the order of study periods, see **Figure 1**. During each study period, participants are admitted to the clinical research unit (CRU). After the measurements in sleep deprived state an interval of at least 5 days is added for recovery.

The measurements will be performed in a standardized order (see **Table 1** for part A and **Table 2** for the measurements of part B).

The total duration of the study for each subject will be up to approximately 50 days for subject who participate in both part A and B.

- Screening: Up to 21 days before first intervention.

- Intervention and study assessments: each study period starts with a urine drug screening and alcohol breath test. Study period 1 (well-rested) starts in the morning upon arrival of the subjects at the CRU. Subjects will perform all assessments twice, in the morning and afternoon. For study period 2 (sleep deprivation), subjects will arrive at the CRU in the evening prior to the study day and stay awake the full night. A single set of the assessments are performed in the morning.

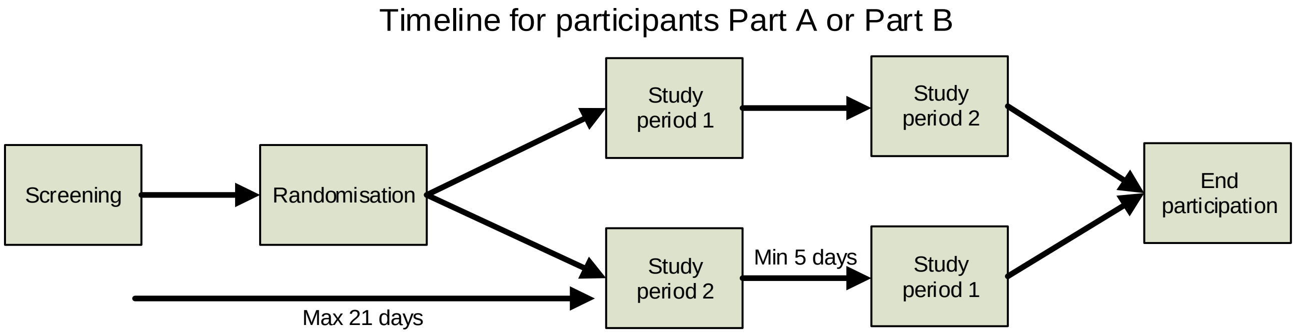

Figure 1: Timeline for participants part A or part B

3.1.1 Screening

The screening phase will only be started after an informed consent has been obtained, according to the Centre for Human Drug Research (CHDR) standard operating procedures (SOP). Screening may take place up to 21 days prior to the first admission to the CRU (e.g. evening before the study night). The entire screening process (including training) will last approximately 4 hours. This includes a medical interview, physical examination, introduction and practice of the assessments applicable to the part of the study.

3.1.2 Intervention and observation period

Subjects will undergo a urine drug screening and alcohol breath test on each arrival at the CRU. During one night per study period they will be deprived from sleep. The subjects will receive standardized meals and non-caffeinated drinks.

Part A
Assessments on all study days will be performed as specified in the Schedule of Assessments (Table 1). The tests will be performed twice in well-rested state, in morning and afternoon, to assess the repeatability.

The first measurement in well/rested state is used for the comparison with sleep deprived state.

Part B
Assessments on all study days will be performed as specified in the Schedule of Assessments (Table 2). The laser stimulation and intra-epidermal stimulation tests will be performed twice in well-rested state, in morning and afternoon, to assess the repeatability.

The first measurement in well/rested state is used for the comparison with sleep deprived state.

4 STUDY POPULATION

4.1 Subject population

Subjects will be recruited via media advertisement or from the subjects' database of the Centre for Human Drug Research, Leiden, the Netherlands.

4.2 Inclusion criteria

Eligible subjects must meet all of the following inclusion criteria:

1. Healthy male subjects, aged 23 to 35 years, inclusive; healthy is defined as no clinically relevant abnormalities identified by a detailed medical and surgical history and a complete physical examination including vital signs.
2. Body mass index (BMI) between 18 and 32 kg/m^2 inclusive.
3. Subjects are active and experienced drivers (applicable for part A only):
    a. In possession of a driver's license, minimum driving experience of 5 years or more.
    b. Minimal car driving mileage of 3000 km per year during the past three years.
4. Able to participate and willing to give written informed consent and to comply with the study restrictions.

4.3 Exclusion criteria

1. History or symptoms of any significant disease including (but not limited to), neurological, psychiatric, endocrine, cardiovascular, respiratory, gastrointestinal, hepatic or renal disorder.
2. Positive test for drugs of abuse at screening or during the study. Positive tests at screening may be repeated.
3. History of and presence of sleep disturbances/disorders.
4. Change in time zones 7 days prior to the study periods.
5. Smoker of more than 10 cigarettes per day prior to screening or who use tobacco products equivalent to more than 10 cigarettes per day.
6. Consume, on average, > 8 units/day of (methyl)-xanthines (e.g. coffee, tea, cola, chocolate) and not able to refrain from use during each stay at the CHDR clinic.
7. Presence of Simulator Sickness Syndrome (applicable for part A only).
8. Subjects indicating pain tests intolerable at screening or achieving tolerance at >80% of maximum input intensity for any pain test for cold, pressure and electrical tests (applicable for part B only).
9. Any circumstances or conditions, which, in the opinion of the investigator, may affect full participation in the study or compliance with the protocol.
10. Dark skin (Fitzpatrick skin type V - VI), wide-spread acne, tattoos or scarring on the volar forearms (applicable for part B only).

4.4 Concomitant medications

All medications (prescription and over-the-counter [OTC]) taken within 30 days of study screening will be recorded.

No prescription medications and OTC medications will be permitted within 14 days prior to study period 1, or less than 5 half-lives (whichever is longer), and during the course of the study. In addition, no vitamin, mineral, herbal, and dietary supplements will be permitted within 7 days prior to study drug administrations, or less than 5 half-lives (whichever is longer), and during the course of the study.

Exceptions will only be made if the rationale is clearly documented by the investigator.

4.5 Lifestyle restrictions

- Approximate meal times will be according to the study schedule.

- The use of (illicit) drugs including cannabis can influence the measurements. Use of drugs (e.g. amphetamines, benzodiazepines, cannabis, cocaine, and opiates) is not permitted from 3 days before and during each study period and until discharge form the study unit.
- Alcohol will not be allowed from at least 24 hours before screening, at each scheduled visit, and whilst in the CRU until discharge. At other times throughout the study, subjects should not consume more than 2 units of alcohol daily on average (one unit is 10 grams of alcohol). Subjects may undergo an alcohol breath test at the discretion of the investigator.
- Subjects will not be allowed to have excessive caffeine consumption, defined as >800 mg per day from 2 days prior to the measurements. Subjects will abstain from caffeine-containing products 4 hours from the screening and start of the occasion until discharge from the study unit. Caffeine quantities defined as: one cup of coffee contains 100 mg of caffeine; one cup of tea, or one glass of cola, or potion of chocolate (dark:100 g, milk 200 g) contains approximately 40 mg of caffeine; one bottle of Red Bull contains approximately 80 mg of caffeine.
- Subjects will abstain from the use of tobacco-or nicotine-containing products (including e-cigarettes and patches) for 24 hours prior to measurements until discharge from the study unit.

4.6 Study drug discontinuation and withdrawal

4.6.1 Study drug interruption or discontinuation

Not applicable

4.6.2 Subject withdrawal

Subjects have the right to withdraw from the study at any time for any reason. Should a subject decide to withdraw from the study, all efforts should be made to complete and report the observations, particularly the follow-up examinations, as thoroughly as possible.

4.6.3 Replacement policy

Subjects withdrawing for reasons other than adverse events or any other tolerability issues with the intervention will be replaced at the discretion of the investigator.

5 INVESTIGATIONAL MEDICINAL PRODUCT

Not Applicable.

6 STUDY ENDPOINTS

6.1 Safety and tolerability endpoints

There are no objective safety and tolerability endpoints. The safety is judged by investigator assessment.

6.2 Pharmacodynamic endpoints

Part A

Simulated and on the road driving

- Standard deviation of lateral position (SDLP) (main parameter).
- Number of lane departures.
- Mean speed (MS).
- Standard deviation of speed (SDS).
- Perceived driving quality scale and perceived effort scale.
- Driving quality scale by driving instructor.
- Car driving exploratory biomarkers (drive safety score, head movements, steering wheel).

Questionnaires

- Leeds Sleep Evaluation Questionnaire (LSEQ), questions 8-14.
- Karolinska sleepiness scale (KSS).

NeuroCart

- Adaptive tracking. Average performance on adaptive tracking CFB (%).
- Body Sway. Total antero-posterior sway (mm).
- Eye movements. percentage time the subjects eyes are in smooth pursuit of the target during smooth pursuit
- VAS Bond and Lader. Composite scores for alertness (mm).

Part B

Questionnaires

- Leeds Sleep Evaluation Questionnaire (LSEQ), questions 8-14.
- Karolinska sleepiness scale (KSS).

NeuroCart

- Adaptive tracking. Average performance on adaptive tracking CFB (%).
- Body Sway. Total antero-posterior sway (mm).
- Eye movements. percentage time the subjects eyes are in smooth pursuit of the target during smooth pursuit
- VAS Bond and Lader. Composite scores for alertness (mm).

EEG

- Resting-EEG. Frequency band power (Hz).
- EEG in combination with auditory steady-state response. The average inter-trial phase coherence between 35 and 45 Hz and between 200 and 500 ms. The average evoked power between 35 and 45 Hz and between 200 and 500 ms.
- EEG in combination with Oddball tasks. P300 and MMN amplitudes (µV) and latencies (msec).
- EEG in combination with visual evoked potentials (VEP). N75 and P100 amplitudes (µV) and latencies (msec).

- EEG in combination with laser-evoked potentials (LEP). Amplitude (μV) and latency (ms) of LEPs (N2, P2, and N2P2).

PainCart

- Heat Pain: Pain Detection Threshold (PDT) (°C)
- Electrical Burst: PDT (mA), Pain Tolerance Threshold (PTT) (mA), Area Under the VAS pain Curve (AUC) (mA*mm), and post-test VAS (mm).
- Electrical Stair (pre-cold pressor): PDT (mA), PTT (mA), Area Under the VAS pain Curve (AUC) (mA*mm), and post-test VAS (mm).
- Electrical Stair (post-cold pressor): PDT (mA), PTT (mA), AUC (mA*mm), and post-test VAS (mm).
- Conditioned Pain Modulation Response (change from electrical stair pre- and post-cold pressor): PDT (mA), PTT (mA), AUC (mA*mm).
- Pressure Pain: PDT (kPa), PTT (kPa), AUC (kPa*mm), and post-test VAS (mm).
- Cold Pressor: PDT (s), PTT (s), Area Above the VAS pain Curve (AAC) (s*mm), and post-test VAS (mm).
- Laser-evoked potentials: Subjective pain perception after LS (LS-NRS): Numeric rating scale (0-10 with 0=no pain & 10=worst pain imaginable).
- McGill Pain Questionnaire for each individual test.

Intra-epidermal electrical stimulation

- Characteristics of the psychophysical curve: nociceptive detection threshold and slope (A50 & β).
- Intra-epidermal electrical stimulation evoked potentials (IESEP).

7 STUDY ASSESSMENTS

See **Table 1** (Part A) or **Table 2** (Part B) for the time points of the assessments.

7.1 Efficacy assessments

On-the-road driving test

Subjects will participate in validated on-road car driving tests. During these tests several safety precautions are taken to guarantee safety of the subjects and regular traffic. A licensed driving instructor (having access to dual controls) guards the safety of the subject and regular traffic during the test. In case the driving instructor or the subject is of the opinion that it is not safe to start or continue the driving test, the test will be interrupted. Subjects will operate a specially instrumented vehicle over a 55-km primary highway circuit, while maintaining a constant speed (95 km/h) and steady lateral position between the delineated boundaries of the right (slower) traffic lane. The subject will be allowed to deviate from this procedure only in order to overtake a slower vehicle traveling in the same traffic lane. The vehicle's speed and lateral position relative to the lane-lines is continuously recorded. These parameters are digitally sampled at 13 Hz by the MobilEye system (MobilEye Vision Technologies Ltd., Israel). Data is edited, off-line, to remove parts where data are disturbed by extraneous events (eg, passing maneuvers). SDLP (cm), ie, the weaving of the car, is the primary outcome variable. SDLP has been shown to be a sensitive measure to demonstrate dose-dependent differences from placebo for alcohol and psychoactive drugs including anxiolytics, antihistamines, antidepressants, hypnotics and analgesics, and drugs of abuse [4, 7, 25]. Standard Deviation of speed (SDS, km/h) will be a secondary variable. Mean lateral position (MLP, +/- cm), and mean speed (MS, km/h) are control variables. During screening, subjects will participate in a training driving test to become acquainted with the procedures of the driving test.

Driving simulator test

Simulated driving sessions will be conducted utilizing the Green Dino driving simulator developed by Green Dino BV, Wageningen, the Netherlands. This computer-based simulator system provides a realistic automotive driving environment, with 3 front channel displays, audio system, 3 pedals (clutch, brake, and gas) manual shift, full-size steering wheel, safety belt, indicators, and hand brake. The controls are linked to a dedicated graphics computer that simulates road environment and dynamic traffic. Subjects will be instructed to drive for 1/2 h with a steady lateral position in the right-lane in a dual-carriageway highway scenario. The driving simulator has been validated by CHDR in a study with subjects dosed with alprazolam or during alcohol clamp (0.5 and 1.0 g/dL; [data on file, CHDR, Leiden]). The SDLP was significantly affected after intravenous administration of both alcohol 0.5 g/L (1.9 cm) and 1.0 g/L (3.0 cm), and after oral administration of 1 mg alprazolam (5.6 cm).

Perceived driving quality scale

Immediately after each driving test, subjects will indicate the perceived quality of their driving performance on a visual analog scale from 0 ('I drove exceptionally poorly') to 20 ('I drove exceptionally well') around a midpoint of 'I drove normally' (see Appendix 1). The level of mental effort they had to invest in performing the task will be assessed on a 15 cm visual analogue scale with markings ranging from 'absolutely no effort' to over 'extreme effort.

Instructor assessment of driving quality

Immediately after each driving test, the driving instructor will indicate the quality of driving performance during the instructor assessment. The assessment is structured with a matrix where aspects of driving receive a score between 1 and 10 (see appendix 2). The indication 'Not Applicable' will be used if there was no assessment possible of the driving aspect. Comments can be applied in the third column. The assessment will be done by the driving instructor and takes approximately 3 minutes.

Karolinska Sleepiness Scale (KSS)
Immediately before and after each driving test, the subject will complete the KSS, a Subject reported assessment used to rate sleepiness on a scale of 1 to 9, ranging from 'extremely alert' (1) to 'very sleepy, great effort to keep awake, fighting sleep (9). See appendix 2 and appendix 3 for an example of the KSS.

Leeds Sleep Evaluation Questionnaire (LSEQ)
Leeds Sleep Evaluation Questionnaire [26] has 10 questions, the answers for which are captured on a VAS scale at the times indicated in the Time and Events Schedule. This clinical tool allows test persons to qualitatively assess their sleep. The LSEQ is electronically adapted in order to measure the correct medication effects. Only questions 8-14 will be analyzed, as questions 1-7 are related to IMP or medication use, which is not applicable in this study.

Adaptive tracking
The adaptive tracking test will be performed as originally described by Borland and Nicholson [27] using customized equipment and software (based on Tracker USB hard-/software (Hobbs, Hertfordshire, UK)). The average performance and the standard deviation of scores over a 3.5-minute period will be used for analysis. This 3.5-minute period is including a run in time of 0.5 minute, in this run in time the data is not recorded. Adaptive tracking is a pursuit-tracking task. A circle moves randomly about a screen. The subject must try to keep a dot inside the moving circle by operating a joystick. If this effort is successful, the speed of the moving circle increases. Conversely, the velocity is reduced if the test subject cannot maintain the dot inside the circle. Each test is preceded by three training sessions and includes two baseline measurements. After 3 practice sessions, learning effects are limited. The adaptive tracking test is more sensitive to impairment of eye-hand co-ordination by drugs than compensatory pursuit tasks or other pursuit tracking tasks, such as the pursuit rotor. The adaptive tracking test has proved to be useful for measurement of CNS effects of alcohol, various other psychoactive drugs and sleep deprivation [1, 28]. The recording of tracking performance will take approximately 5 minutes.

Body Sway
The body sway meter allows measurement of body movements in a single plane, providing a measure of postural stability. Body sway is measured with a pot string meter (celesco) based on the Wright ataxia meter [29]. At CHDR, the method has been used to demonstrate effects of sleep deprivation [2], alcohol [30], benzodiazepines [2, 31] and other psychoactive agents (data on file). With a string attached to the waist, all body movements over a period of time are integrated and expressed as mm sway. Subjects will be instructed to wear a pair of comfortable, low-heeled shoes on each session. Before starting a measurement, subjects will be asked to stand still and comfortable, with their feet approximately 10 cm part and their hands in a relaxed position alongside the body and eyes closed. Subjects may not talk during the measurement. The total period of body-sway measurement will be two minutes.

Saccadic eye movements
Saccadic peak velocity is one of the most sensitive parameters for sedation [2, 28, 32]. The use of a computer for measurement of saccadic eye movements was originally described by Baloh et al. [33], and has been validated at CHDR by Van Steveninck et al. [28, 32]. While the sedative effects of 20 mg oral temazepam were detectable by subject self-report, visual analogue scales and SEM testing, at a dose of 5 mg were only detectable with measures of SEM [34]. The effects of 1 night of sleep deprivation (suggested as a threshold level of clinically significant sedation) were consistently detectable by SEM with a sustained decrease in saccadic peak velocity of 9 to 10% observed [2]. Saccadic eye movements will be recorded at a training session, pre-dose and at times specified in the protocol. Recording of eye movements will be performed in a quiet room with dimmed lightning. There will be only one subject per session in the same room.

Recording and analysis of saccadic eye movements is conducted with a microcomputer-based system for sampling and analysis of eye movements. The program for signal collection and the AD-converter is from Cambridge Electronic Design (CED Ltd., Cambridge, UK), the amplification by Grass (Grass-Telefactor, An Astro-Med, Inc. Product Group, Braintree, USA) and the sampling and analysis scripts are developed at the CHDR (Leiden, the Netherlands). Disposable silver-silver chloride electrodes (Ambu Blue Sensor N) will be applied on the forehead and beside the lateral canthi of both eyes of the subject for registration of the electro-oculographic signals. Skin resistance is reduced to less than 5 kOhm before measurements. Head movements are restrained using a fixed head support. The target consists of a moving dot that is displayed on a computer screen. This screen is fixed at 58 cm in front of the head support.
Saccadic eye movements are recorded for stimulus amplitudes of approximately 15 degrees to either side. Fifteen saccades are recorded with interstimulus intervals varying randomly between 3 and 6 seconds. Average values of latency (reaction time), saccadic peak velocity of all correct saccades and inaccuracy of all saccades will be used as parameters. Saccadic inaccuracy is calculated as the absolute value of the difference between the stimulus angle and the corresponding saccade, expressed as a percentage of the stimulus angle.

Smooth pursuit eye movements
The same system as used for saccadic eye movements is also used for measurement of smooth pursuit. For smooth pursuit eye movements, the target moves at a frequency ranging from 0.3 to 1.1 Hz, by steps of 0.1 Hz. The amplitude of target displacement corresponds to 22.5 degrees eyeball rotation to both sides. Four cycles are recorded for each stimulus frequency. The method has been validated at CHDR by Van Steveninck et al. [32] based on the work of Bittencourt et al. [34] and the original description of Baloh et al. [33]. The time in which the eyes are in smooth pursuit of the target will be calculated for each frequency and expressed as a percentage of stimulus duration. The average percentage of smooth pursuit for all stimulus frequencies will be used as parameter. The measurements of smooth pursuit eye movements will take approximately 3 minutes.

VAS Bond and Lader
VAS as originally described by Norris have often been used previously to quantify subjective effects of a variety of sedative agents [35, 36]. Dutch versions of the scales have been frequently employed at CHDR for a variety of sedative agents [28] and circumstances [36]. At each time point of assessment the subject indicates (with a mouse click on the computer screen) on 16 horizontal VAS how he/she feels. The 16 measurements are recorded in the subject's eCRF. From these measurements, three main factors are calculated as described by Bond and Lader [37]: alertness (from nine scores), mood (from five scores), and calmness (from two scores). The VAS are provided in Appendix 4 (Dutch version) and Appendix 5 (original English version). Completing the set of VAS will take approximately 2 minutes.

EEG measurements
EEG is continuously recorded using a 40-channel recording system (Refa-40, TMSi B.V., the Netherlands). Electrodes are placed according to the international 10-20 system with a total of 21 leads (MFi B.V.), but replacing electrodes placed at the earlobes (i.e., A1 and A2) with electrodes placed at the mastoids (i.e., M1 and M2). The IES and LEP tasks will use 32 leads EEG. The scalp electrode impedance are kept below 5kΩ. The ground electrode is placed at AFz. Additionally, to detect ocular artefacts, vertical and horizontal EOG are recorded. Two Ag/AgCl electrodes are placed at the outer canthi of both eyes, and two Ag/AgCl electrodes are placed approximately 2 cm above and below the right eye. All signals are sampled at a sampling rate of 1024 Hz and are filtered prior to storage using a first order recursive high-pass filter with a cut-off frequency at 0.1 Hz. Digital markers are recorded by the amplifier indicating the onset of each stimulus.

Resting EEG ask description

Resting-state EEG recordings with open and closed eyes for 5 min in each eye state will be performed [38]. Each recording employs alternating periods with eyes open and closed with a duration of 64-seconds for each period. Subjects face a featureless wall and are instructed not to stare, not to move their head and eyes, and to suppress eye blinks.

Auditory steady state response task description
Auditory Steady State Response (ASSR) recordings will be performed. Subjects are seated in a comfortable chair and instructed to sit still, relax, and to close their eyes. They are also instructed to not pay special attention to auditory tones. Auditory stimuli are presented through headphones at a sound pressure level of 65 dB+- 2 dB. Each stimulus is a 500ms burst of 1ms monophasic rectangular pulses at 40 Hz. The inter-stimulus interval is 700ms with no onset asynchrony.

Active auditory oddball task description
Subjects are seated with EEG cap and headphones on and are instructed to sit still and relax. During the task, subjects are being presented auditory tones. Subjects are to pay attention to the tones and press a response-button when they hear an infrequent/deviant tone. A total of 500 tones are presented of which 400 are presented as frequent stimuli and 100 as deviant/infrequent stimuli. Therefore, infrequent tones have a probability of 0.2. The first five trials are frequent tones and there are at least two frequent tones between two deviant/infrequent tones. The frequent and infrequent tones are 150ms tones of 1000 Hz and 500 Hz at a sound pressure level of 75dB, respectively. All tones have a 5ms rise and fall time. Tones are presented at a fixed rate of 1 Hz.

Passive auditory oddball task description
Subjects are seated with EEG cap and headphones on and are instructed to sit still and relax. During the task, subjects are watching a silent movie while being presented auditory tones. Subjects are not to pay special attention to the tones. A total of 750 tones are presented of which 600 are presented as frequent stimuli and 150 as deviant/infrequent stimuli. Therefore, infrequent tones have a probability of 0.2. The first five trials are frequent tones and there are at least two frequent tones between two deviant/infrequent tones. The frequent and infrequent tones are 150ms tones of 1000 Hz and 1200 Hz at a sound pressure level of 80dB, respectively. All tones have a 5ms rise and fall time. Tones are presented at a fixed rate of 2 Hz.

Visual evoked potentials task description
Visual Evoked Potentials (VEPs) recordings will be performed. Subjects are seated in a comfortable chair while resting their head in a chin-rest mounted 58 cm from a screen. Subjects are instructed to sit still and watch a red cross on the screen. Visual stimuli are presented on a screen with a contrast higher than 80 [%] and a mean luminance of 50 [cd / m^2]. The stimuli are two types of checkerboard-patterns: 1.00 degrees width and 0.25 degrees width. There are a total of 320 phase reversals for each type of stimulus at a rate of 2 [Hz].

Intra-epidermal stimulation (IES)
An electrode specifically designed for nociceptive stimulation [39] is placed on the volar forearm. The settings of the IES for two types of stimuli are given in **Table 3**:

Table 3: Settings for IES

	**Type I**	**Type II**
**Shape of pulse**	Square	Square
**Pulse width (us)**	210	210
**Number of pulses (#)**	1	2
**Inter-pulse interval (ms)**	NA	10
**Inter-stimulus interval (s)**	2.5-3.5	2.5-3.5

The adaptive staircase procedure method by Doll et al. [40] will be used to estimate the detection thresholds and slopes of IES. Stimulating below twice the detection threshold will ensure preferential stimulation of Aδ fibres (nociceptive system) [41].
Subjects are asked to release a push button when a stimulation is perceived. If the stimulation has not been perceived the adaptive staircase procedure will generate a new stimulus and the stimulation will be determined as not detected. The push button will also measure the reaction time between stimulation and release for IES.
Endpoints for both stimulus types:

- Detection thresholds (mA)
- Slope (mA-1)
- Rate of detection (%)
- Reaction time (ms)

These settings are in accordance with literature [41-44].
Baseline is defined as the value after a normal night of sleep.

Laser Stimulation (LS)
LS will be performed by the STIMUL 1340 Nd:YAP laser system. The settings of the laser will be:

- Stimulus duration: 5 ms
- Laser diameter: 5 mm
- Energy: 2 J
- Energy density: 102 mJ/mm2
- Inter-stimulus time: 6-8 s (randomized)
- For the LS assessments, only reaction time will be evaluated as psychophysical parameter during the tests. Subjects are asked to

Endpoint:

- Reaction time (ms)

The electrophysiological parameters will be the same as in the IES assessments. Due to the low signal to noise ratio (SNR) of EEG data, at least 15-40 stimulations are needed to find reliable LEP values [45, 46]. Therefore, in this study 20 stimulations will be used per location per stimulus block. EEG data will be collected with a cap using the 10-20 system. Artefacts due to eye blinking, etc. will be removed from the analysis.
The raw data will be analysed using FieldTrip by Matlab, giving the following:
Endpoints (in primary, secondary16 and control area):

- Amplitude (μV) of N1, N2, P1, P2, N2P2 peaks
- Latency (ms) of N1, N2, P1, P2, N2P2 peaks

These settings are in accordance with literature [45, 47, 48].
Baseline is defined as the value after a normal night of sleep.

PainCart assessments
Nociceptive (pain) detection and tolerance thresholds will be measured using the PainCart, an integrated range of tests for measuring different modalities of nociception. The PainCart aims to assess as objectively as possible the levels of pain induced in human subjects by a variety of potentially noxious stimuli. All measurements will be performed in a quiet room with ambient illumination and temperature. During each testing session, there will only be 1 subject per room. During nociceptive tests, subjects will either be sitting or resting comfortably on a bed (high-Fowler's position, with knees supported).

Pain intensity will be measured continuously for each of the following nociceptive tests with subjects rating their pain intensity using a 100% electronic visual analog scale (eVAS)-slider, with 0 and 100

defined as 'no pain' and 'worst pain tolerable', respectively. The equipment is programmed to cease giving stimuli if pain intensity reaches the maximum possible score. Data from the eVAS will inform the PDT (the start of pain, when a subject moves the eVAS slider away from 0) and the PTT (when a subject moves the eVAS slider to the end point, triggering a stop for any particular pain test). In addition, the area under the VAS pain curve and/or post-test VAS may be determined for any of the pain tests performed in the study. The following tests will be performed:

Electrical Stimulation Pain Test (Electrical Stair and Burst)
For cutaneous electrical pain, 2 electrodes (Ag-AgCl) will be placed on clean (scrubbed) skin overlying the left tibial bone 100 mm distal from the caudal end of the patella (middle of the first electrode is placed 100 mm distal the caudal end of the patella and middle of the second electrode 135 mm directly underneath the first). Electrical resistance between electrodes will be less than 2 kΩ [49].
For single (stair) stimulus, each stimulus (10-Hz tetanic pulse with a duration of 0.2 ms) will be controlled by a computer-controlled constant current stimulator. Current intensity will be increased from 0 mA in steps of 0.5 mA·s-1 (cutoff 50 mA). The pain intensity after each stimulation will be measured using the eVAS, until pain tolerance level is reached or a maximum of 50 mA is reached. [49] To measure conditioned pain modulation (formerly diffuse noxious inhibitory control), the single stimulus will be repeated within 5 minutes of the end of the cold pressor task.
For repeated (burst) stimulus, each single stimulus is repeated 5 times with a frequency of 2 Hz. Pain threshold is taken as the value (mA) whereby a subject indicates either: all 5 stimuli are painful, or the train of 5 stimuli started feeling non-painful but ends feeling painful (VAS > 0) (Adapted from the method of Arendt-Nielsen et al, [50].

Pressure Pain Test
This method of pressure pain induction has been shown to primarily assess nociception generated from the muscle with minimal contribution by cutaneous nociceptors [51] and is based on methods previously described [52]. Briefly, an 11 cm wide tourniquet cuff (VBM Medizintechnik GmbH, Sulz, Germany) will be placed over the gastrocnemius muscle with a constant pressure rate increase of 0.5 kPa/s controlled by an electro-pneumatic regulator (ITV1030-31F2N3-Q, SMC Corporation, Tokyo, Japan),Power1401mkII analogue–to-digital converter and Spike2 software (CED, Cambridge, UK). The subject will sit comfortably with their foot flat on the floor and rate their pain intensity using the eVAS, with 0 and 100 defined as "no pain" and "worst pain tolerable", respectively. The pneumatic pressure will be increased until the subject indicates their PTT, or a maximum pressure of 100 kPa is achieved, at which point the device releases pressure to the tourniquet.

Cold Pressor Pain Test
The method of cold pressor pain is based on the methods previously described [53, 54]. Subjects place their non-dominant hand into a water bath (minimal depth 200 mm) at 35 ± 0.5 °C for 2 minutes. At 1 minute 45 seconds a blood pressure cuff on the upper-arm will be inflated to 20 mmHg below resting diastolic pressure. At 2 minutes the subject will then move their hand from the warm water bath, directly placing their hand into a similar sized bath at 1.0 ± 0.5 °C. The subjects will be instructed to indicate when pain detection threshold is reached (first change in sensation from cold non-painful to painful) as well as the increase in pain intensity, by moving the eVAS slider. When pain tolerance is reached (sensation is no longer tolerable; eVAS slider at 100 mm), or when a time limit (120 s) is reached, subjects are instructed to remove their arm from the water, at which point the blood pressure cuff will deflate. Analysis of the recorded data results in the following parameters for cold pressor pain measurements are: Time from cold water immersion until pain detection threshold (seconds), pain tolerance threshold (seconds), and area above the curve (AC) (seconds*mm).

Thermal Pain Tests
To determine heat PDTs in normal skin as well as primary hyperalgesia to heat, thermal PDTs will be measured with a thermode (Medoc Q-Sense) with a contact area of 30 mm x 30 mm. The thermode

will be placed in the middle of the control or primary area on the subject's volar forearm, starting with the control. The initial temperature of the thermode will be 32°C and will increase by 0.5°C·per second until the subject perceives the stimulus as painful, based on PDTs, or when a temperature of 50°C is reached. The average of a triplicate measurement will be used to determine PDTs.

McGill Questionnaire

McGill's Pain Questionnaire (MPQ-DLV), [55-57] a classical pain questionnaire, which addresses the multiple dimensions of pain: sensory (temporal, spatial and descriptive), affective (autonomic and anxiety), evaluative and miscellaneous. The questionnaire consists of general pain questions (duration, location, radiation, and frequency), a pain word list (divided in twelve sensory, five affective, and three evaluative adjectives) and 10 cm visual analogue lines of pain intensity (current and minimum-maximum). The scoring algorithm sums the number and the rank orders of adjectives within each dimension, and measures the different visual analogue scores [55]. The McGill is particularly used to assess how patients perceive pain. The momentary 10-cm visual analogue lines alone, ranging from 0 ('no pain') to 100 mm ('worst pain conceivable') are well suited for repeated 'pharmacodynamic' pain measurements.

Laboratory parameters

The following samples will be collected for the following clinical laboratory tests:

Lab	Tests	Collection & Analysis
Alcohol	Alcohol Breath Test	The hand-held Alco-Sensor IV meter (Honac, Apeldoorn, the Netherlands) will be used to measure the breath ethanol concentrations.
Urine drug screen	Cocaine, amphetamines, opiates (morphine), benzodiazepines and cannabinoids.	A urine specimen will be analysed at CHDR by test kit (InstAlert, Innovacon, San Diego, USA).

7.2 Safety and tolerability assessments

The definitions, reporting and follow-up of AEs, SAEs and potential pregnancies are described in section 8. There are no safety and tolerability assessments included in this study besides the investigator assessment during the driving test as well as the observation of adverse events.

7.2.1 Investigator assessment during driving test

Part A

There is a safety assessment in place to ensure the safety of the subjects. Before driving the subjects are instructed about the possibility to terminate the on-the-road driving test. The driving instructor will emphasize the possibility for early termination of the driving test. The test can also be prematurely stopped by the driving instructor when needed to ensure the safety of the subjects and other road-users.

8 SAFETY REPORTING

8.1 Definitions of adverse events

An Adverse Event (AE) is any untoward medical occurrence in a subject who is participating in a clinical study performed. The AE does not necessarily have to follow the administration of a study drug, or to have a causal relationship with the study drug. An AE can therefore be any unfavourable and unintended sign (including an abnormal laboratory or vital sign finding), symptom, or disease temporally associated with the study participation, whether or not it is related to the study drug.

8.1.1 Intensity of adverse events

The intensity of clinical AEs is graded three-point scale as defined below:

- Mild: discomfort noticed but no disruption of normal daily activity;
- Moderate: discomfort sufficient to reduce or affect normal daily activity;
- Severe: inability to work or perform daily activity.

8.1.2 Chronicity of adverse events

The chronicity of the AE will be classified by the investigator on a three-item scale as defined below:

- Single occasion: single event with limited duration;
- Intermittent: several episodes of an event, each of limited duration;
- Persistent: event which remained indefinitely.

8.1.3 Action

Eventual actions taken will be recorded.

8.1.4 Serious adverse events

A Serious Adverse Event (SAE) is defined by the International Conference on Harmonization (ICH) guidelines as any AE fulfilling at least one of the following criteria:

- results in death;
- is life threatening (at the time of the event);
- requires hospitalisation or prolongation of existing inpatients' hospitalisation;
- results in persistent or significant disability or incapacity;
- is a congenital anomaly or birth defect; or
- any other important medical event that did not result in any of the outcomes listed above due to medical or surgical intervention but could have been based upon appropriate judgement by the investigator.

An elective hospital admission will not be considered as a SAE.

8.1.5 Reporting of serious adverse events

SAEs will be reported according to the following procedure.

The investigator will report the SAEs through the web portal ToetsingOnline (see https://toetsingonline.nl/) to the accredited EC that approved the protocol, within 7 days of first knowledge for SAEs that result in death or are life threatening followed by a period of maximum of 8 days to complete the initial preliminary report. All other SAEs will be reported within a period of maximum 15 days after the sponsor has first knowledge of the SAE.

8.1.6 Follow-up of adverse events
All AEs will be followed until they have abated or until a stable situation has been reached. Depending on the event, followup may require additional tests or medical procedures as indicated, and/or referral to the general physician or a medical specialist.

8.2 Temporary halt for reasons of subject safety
In accordance to section 10, subsection 4, of the WMO, the investigator will inform the subjects and the EC if anything occurs, on the basis of which it appears that the disadvantages of participation may be significantly greater than was foreseen in the research proposal. The study will be suspended pending further review by the EC, except insofar as suspension would jeopardise the subjects' health. The investigator will ensure that all subjects are kept informed.

9 STATISTICAL METHODOLOGY AND ANALYSES

9.1 Statistical analysis plan

All safety and statistical programming is conducted with SAS 9.4 for Windows or newer (SAS Institute Inc., Cary, NC, USA). A Statistical Analysis Plan (SAP) will be written and finalized before the study closure, i.e., database closure. The SAP will provide full details of the analyses, the data displays and the algorithms to be used for data derivations.

9.2 Protocol violations/deviations

Protocol deviations will be identified based on conditions related to the categories below:

- Protocol entry criteria
- Forbidden concomitant medications
- Missing evaluations for relevant endpoints
- Other protocol deviations occurring during study conduct.

Major protocol deviations will be identified before the study closure, and listed where appropriate.

9.3 Power calculation

Part A

For this power calculation we used aggregated data from a previous CHDR study (CHDR1429) in order to calculate the intra- and inter- SD. The variabilities are based on the change from SDLP values.
For a simulator driving test, a sample size of 24 will have a power of 0.80 to detect a difference in means of 3.88 cm, assuming a standard deviation of differences of 6.5 cm, using a paired t-test with a 0.05 two-sided significance level.

Part B

For this power calculation we used aggregated data from previous CHDR studies (CHDR1311, CHDR1422, CHDR1425, CHDR1431 and CHDR1440) in order to calculate the intra- and inter- SD. The variabilities are based on the change from baseline cold pressure pain test (coldPTT) values. For cold pressor pain test, a sample size of 24 will have a power of 0.80 to detect a difference in means of 2.09 (sec), assuming a standard deviation of differences of 3.5 (sec), using a paired t-test with a 0.050 two-sided significance level.

9.4 Missing, unused and spurious data

All missing or incomplete safety and pharmacodynamics (PD) data, including dates and times, are treated as such. Missing test results or assessments will not be imputed. Missing PD data, indicated as ‘M’ in the data listing, will be estimated within the statistical mixed model using SAS PROC MIXED.

Part A

Prematurely stopped on-the-road tests will only be included if more than 20 kilometres is driven, and only the last 10 kilometres will be used for data analysis.

The handling of missing, unused and spurious data will be documented in the study report.

9.5 Analysis sets

Data of all subjects participating in the study will be included in the analyses if the data can meaningfully contribute to the objectives of the study.

9.5.1 Safety set

The safety population will be defined as all subjects who were validated and received at least 1 study intervention.

9.5.2 Pharmacodynamic analysis set

The analysis population for efficacy is defined as all subjects who were validated, received at least one intervention, and have at least one post-baseline assessment of the parameter being analysed.

9.6 Subject disposition

Subject disposition will be listed by subject.

The following subject data will be summarized:

- number and percentage of subjects screened,
- number and percentage of subjects enrolled,
- number and percentage of subjects completed or prematurely discontinued;
- number and percentage of subjects included in safety population;
- number of subjects included in the PS analysis population;
- number and percentage of subjects completed.

A subject who completed the study is defined as a subject where the last assessment is finished.

9.7 Baseline parameters and concomitant medications

9.7.1 Demographics and baseline variables

Continuous demographic variables (e.g., age, height, weight, BMI) will be summarized by descriptive statistics (n, mean, SD, median, Min, Max).

Qualitative demographic characteristics (sex, race/ethnicity) will be summarized by counts and percentages.

9.7.2 Medical history

Medical history will only be listed.

9.8 Safety and tolerability endpoints

The safety set is used to perform all safety analyses.

9.8.1 Adverse events

The AE coding dictionary for this study will be Medical Dictionary for Regulatory Activities (MedDRA). It will be used to summarize AEs by primary system organ class (SOC) and preferred term (PT).
All adverse events will be displayed in listings.

9.8.1 Prematurely discontinued on-the-road driving tests

Before and during the on-the-road driving test the subject and/or the driving instructor can stop the test prematurely for safety reasons. This will be noted as an event and the events will be summarized.

The number of subjects who did not finish the test will be summarized by:

1. Person who stopped the test (subject or driving instructor);
2. Amount of kilometres driven (rounded to 5km);

9.9 Pharmacodynamics endpoints

The analysis of part A and part B will done separately. The measurements of the afternoon after normal sleep will not be used for the primary analysis. The repeatability of the parameters measured during morning and during the afternoon after normal sleep will be assessed separately on the subset of the after normal sleep data.

The final analysis will be preceded by a data review which consists of individual graphs per visit by time of all pharmacodynamic measurements by time. The graphs will be used to detect outliers and measurements unsuitable for analysis.

The PD parameters will be listed by subject, group (sleep/sleep deprivation), visit and time. Individual graphs by visit/time will be generated.

All PD endpoints will be summarised (n, mean, SD, SEM, median, Min and Max values) by group (sleep/sleep deprivation), and will be presented graphically per group/time in bar graphs with standard deviation as error bars.

Parameters will initially be analysed without transformation, but if the data suggest otherwise, log-transformation may be applied. Log-transformed parameters will be back-transformed after analysis where the results may be interpreted as percentage change.

To estimate the differences between groups (sleep deprived/sleep) data will be analysed with a mixed model analysis of variance with fixed factor group and random factor subject. The contrast sleep vs sleep deprived will be calculated within the model.

To estimate inter- and intra-subject variability the subset data of the sleep group will be analysed with a mixed model analysis of variance with fixed factor time (morning/afternoon) and random factor subject. The variances will be estimated within the model.

The Kenward-Roger approximation will be used to estimate denominator degrees of freedom and model parameters will be estimated using the restricted maximum likelihood method.

Correlations of the change between sleep and sleep deprived values between the cognitive tests, simulated and on-the-road driving will be calculated.

The intra-subject variabilities will be reported as coefficient of variation (CV).

9.10 Exploratory analyses and deviations

Exploratory data-driven analyses can be performed with the caveat that any statistical inference will not have any confirmatory value.
Deviations from the original statistical plan will be documented in the clinical study report.

10 GOOD CLINICAL PRACTICE, ETHICS AND ADMINISTRATIVE PROCEDURES

10.1 Good clinical practice

10.1.1 Ethics and good clinical practice

The investigator will ensure that this study is conducted in full compliance with the protocol, the principles of the Declaration of Helsinki (www.wma.net), ICH GCP guidelines (http://www.ich.org/products/guidelines.html), and with the laws and regulations of the country in which the clinical research is conducted.

10.1.2 Ethics committee / institutional review board

The investigator will submit this protocol and any related documents to an Ethics Committee (EC) and the Competent Authority (CA). Approval from the EC and the statement of no objection from the CA must be obtained before starting the study, and should be documented in a dated letter/email to the investigator, clearly identifying the trial, the documents reviewed and the date of approval. A list of EC members must be provided, including the functions of these members. If study staff were present, it must be clear that none of these persons voted.

Modifications made to the protocol after receipt of the EC approval must also be submitted as amendments by the investigator to the EC in accordance with local procedures and regulations.

10.1.3 Informed consent

It is the responsibility of the investigator to obtain written informed consent from each individual participating in this study after adequate explanation of the aims, methods, objectives and potential hazards of the study. The investigator must also explain to the subjects that they are completely free to refuse to enter the study or to withdraw from it at any time for any reason.

The Informed Consent and Subject Information will be provided in Dutch.

10.1.4 Insurance

The investigator has a liability insurance which is in accordance with article 7, subsection 6 of the WMO.
The investigator (also) has an insurance which is in accordance with the legal requirements in the Netherlands (Article 7 WMO and the Measure regarding Compulsory Insurance for Clinical Research in Humans of 23rd June 2003). This insurance provides cover for damage to research subjects through injury or death caused by the study.

- € 650,000.- (i.e., six hundred and fifty thousand Euro) for death or injury for each subject who participates in the Research;
- € 5,000,000.- (i.e., five million Euro) for death or injury for all subjects who participate in the Research;
- € 7,500,000.- (i.e., seven million and five hundred thousand Euro) for the total damage incurred by the organisation for all damage disclosed by scientific research for the Sponsor as 'verrichter' in the meaning of said Act in each year of insurance coverage.

The insurance applies to the damage that becomes apparent during the study or within 4 years after the end of the study.

For the on-road driving test specifically, an additional insurance has been taken out. The following items are covered:

- WA personenauto; insured value for damage to property: € 1.220.000,-
- WA personenauto; insured value for damage to persons:: € 6.070.000,-
- Casco personenauto; 'hull car insurance'; insured value: € 34.162,-
- SVI (schadeverzekering voor inzittenden); insurance for damage to passengers; insured value: € 500.000,-

10.2 Study funding

CHDR is the sponsor of the study and is funding the study.

10.3 Data handling and record keeping

10.3.1 Data collection

Data will be recorded on electronic data collection forms in Promasys for subsequent tabulation and statistical analysis. The data will be handled confidentially.
A Subject Screening and Enrolment Log will be completed for all eligible or non-eligible subjects with the reasons for exclusion.

10.3.2 Database management and quality control

All data from paper source will be entered into the Promasys database twice, by two different individuals. A quality control check will be done by CHDR staff on all data entered in the Promasys database, using data entry progress checks and database listings (blind data review). Errors with obvious corrections will be corrected before database lock.
Results of computer (NeuroCart/PainCart/ECG ect.) tests and electronically captured questionnaires, will be sent electronically to CHDR and loaded into the database.
After the database has been declared complete and accurate, the database will be locked. Any changes to the database after that time can only be made by joint written agreement between the investigator and the statistician.

10.4 Access to source data and documents

All study data will be handled confidential. The investigator will retain the originals of all source documents generated at CHDR for a period of 2 years after the report of the study has been finalised, after which all study-related documents will be archived (at a minimum) on micro-film which will be kept according to GCP regulations. After 2 years the sponsor will be notified that the source documents can be retained with the sponsor or destroyed.
The investigator will permit trial-related monitoring, audits, EC review and regulatory inspections, providing direct access to source data and documents.

10.5 Quality control and quality assurance

This study will be conducted according to applicable Standard Operating Procedures (SOPs). Quality assurance will be performed under the responsibility of CHDR's Quality Assurance manager.

10.5.1 Monitoring

An initiation visit will be performed before the first subject is included. Monitoring visits and contacts will occur at regular intervals thereafter, according to a frequency defined in the study-specific monitoring plan. A close-out visit will be performed after study closure.

10.6 Protocol amendments

Any change to a protocol has to be considered as an amendment.

10.6.1 Substantial amendment

Significant changes that affect subject safety and/or the scientific value of a trial require a substantial amendment. Examples of significant changes are given in EU guidelines on the request to the competent authorities for authorisation of a clinical trial on a medicinal product for human use, the notification of substantial amendments and the declaration of the end of the trial (CT-1, 2010/C 82/01). The need for submitting a substantial amendment is the responsibility of the sponsor. Substantial amendments are to be approved by the appropriate EC and the CA will need to provide a 'no grounds for non-acceptance' notification prior to the implementation of the substantial amendment.

10.6.2 Non-substantial amendment

Non substantial amendments do not affect subject safety or the scientific integrity of the trial. Non-substantial amendments will be approved (signed) by the investigator(s) and will be recorded and filed by the investigator. Non-substantial amendments will be submitted to the EC for information only.

The CA will only be notified by changes in Eudract form and ABR form (if applicable) at toetsingonline. The implementation of a non-substantial amendment can be done immediately.

The EU guideline CT-1 2010/C 82/01 stipulates the importance of preventing over-reporting. Therefore the following changes are by definition non-substantial in this study:

- changes in assay-type and / or institution where an assay will be performed, provided that validated assays will be used;
- editorial changes to documents in the submission dossier including the volunteer information sheets and the protocol. An editorial change is defined as a modification in the documents of typographical errors and other modifications that in no way alter the meaning or content of the document
- determination of additional parameters in already collected materials, which are in agreement with the study objectives and do not provide prognostic or genetic information;
- other statistical analyses than described in the protocol.
- A change in clinical staff, including the principal investigator, when this concerns regular staff members of CHDR who comply with internal regulations for training and authorisation.

10.6.3 Urgent amendment

An urgent amendment might become necessary to preserve the safety of the subjects included in the study. The requirements for approval should in no way prevent any immediate action being taken by the investigators in the best interests of the subjects. Therefore, if deemed necessary, an investigator can implement an immediate change to the protocol for safety reasons. This means that, exceptionally, the implementation of urgent amendments will occur before submission to and approval by the EC(s) and CA.

10.7 End of study report

The investigator will notify the EC of the end of the study within a period of 8 weeks. The end of the study is defined as the last subject's last visit. In case the study is ended prematurely, the investigator will notify the EC within 15 days, including the reasons for the premature termination.

Within one year after the end of the study, the investigator will submit a final study report with the results of the study, including any publications/abstracts of the study, to the EC. The principal investigator will be the signatories for the study report.

10.8 Public disclosure and publication policy

In accordance with standard editorial and ethical practice, the results of the study will be published, if applicable. The authorship guidelines of the Vancouver Protocol[1] will be followed regarding co-authorship.

[1] http://www.icmje.org/

11 STRUCTURED RISK ANALYSIS

Not applicable.

12 REFERENCES

1. Groeneveld, G.J., J.L. Hay, and J.M. Van Gerven, *Measuring blood-brain barrier penetration using the NeuroCart, a CNS test battery.* Drug Discov Today Technol, 2016. **20**: p. 27-34.
2. van Steveninck, A.L., et al., *The sensitivity of pharmacodynamic tests for the central nervous system effects of drugs on the effects of sleep deprivation.* J Psychopharmacol, 1999. **13**(1): p. 10-7.
3. Ramaekers, J.G., *Drugs and Driving Research in Medicinal Drug Development.* Trends Pharmacol Sci, 2017. **38**(4): p. 319-321.
4. Verster, J.C., et al., *Prolonged nocturnal driving can be as dangerous as severe alcohol-impaired driving.* J Sleep Res, 2011. **20**(4): p. 585-8.
5. Berthelon, C. and G. Gineyt, *Effects of alcohol on automated and controlled driving performances.* Psychopharmacology (Berl), 2014. **231**(10): p. 2087-95.
6. O'Hanlon, J.F., *Driving performance under the influence of drugs: rationale for, and application of, a new test.* Br J Clin Pharmacol, 1984. **18 Suppl 1**: p. 121S-129S.
7. Verster, J.C. and T. Roth, *Standard operation procedures for conducting the on-the-road driving test, and measurement of the standard deviation of lateral position (SDLP).* Int J Gen Med, 2011. **4**: p. 359-71.
8. Verster, J.C., et al., *Lapses of attention as outcome measure of the on-the-road driving test.* Psychopharmacology (Berl), 2014. **231**(1): p. 283-92.
9. Verster, J.C. and T. Roth, *Effects of central nervous system drugs on driving: speed variability versus standard deviation of lateral position as outcome measure of the on-the-road driving test.* Hum Psychopharmacol, 2014. **29**(1): p. 19-24.
10. Helland, A., et al., *Comparison of driving simulator performance with real driving after alcohol intake: a randomised, single blind, placebo-controlled, cross-over trial.* Accid Anal Prev, 2013. **53**: p. 9-16.
11. FDA, *Evaluating Drug Effects on the Ability to Operate a Motor Vehicle - Guidance for Industry*, C.f.D.E.a. Research, Editor. 2017.
12. Huizinga, *Suitability of simulated driving in comparison to laboratory-based tests (NeuroCart) to assess the pharmacodynamics of alprazolam and alcohol.* J Psychoprahmacology.
13. Jacobs, M., *Altered driving performance of symptomatic Huntington's disease gene carriers in simulated road conditions.* Traffic Injury Prevention (accepted), 2018.
14. Ferreira, C., et al., *Electroencephalographic changes after one night of sleep deprivation.* Arq Neuropsiquiatr, 2006. **64**(2B): p. 388-93.
15. Raz, A., L.Y. Deouell, and S. Bentin, *Is pre-attentive processing compromised by prolonged wakefulness? Effects of total sleep deprivation on the mismatch negativity.* Psychophysiology, 2001. **38**(5): p. 787-95.
16. Ramautar, J.R., et al., *Coupling of infraslow fluctuations in autonomic and central vigilance markers: skin temperature, EEG beta power and ERP P300 latency.* Int J Psychophysiol, 2013. **89**(2): p. 158-64.
17. Schuh-Hofer, S., et al., *One night of total sleep deprivation promotes a state of generalized hyperalgesia: a surrogate pain model to study the relationship of insomnia and pain.* Pain, 2013. **154**(9): p. 1613-21.
18. Smith, M.T., Jr., et al., *Sex differences in measures of central sensitization and pain sensitivity to experimental sleep disruption: Implications for sex differences in chronic pain.* Sleep, 2018.
19. Karmann, A.J., B. Kundermann, and S. Lautenbacher, *[Sleep deprivation and pain: a review of the newest literature].* Schmerz, 2014. **28**(2): p. 141-6.
20. Odegard, S.S., et al., *The effect of sleep restriction on laser evoked potentials, thermal sensory and pain thresholds and suprathreshold pain in healthy subjects.* Clin Neurophysiol, 2015. **126**(10): p. 1979-87.
21. Lautenbacher, S., B. Kundermann, and J.C. Krieg, *Sleep deprivation and pain perception.* Sleep Med Rev, 2006. **10**(5): p. 357-69.
22. Matre, D., et al., *Conditioned pain modulation is not decreased after partial sleep restriction.* Eur J Pain, 2016. **20**(3): p. 408-16.

23. Pieh, C., et al., *Night-shift work increases cold pain perception.* Sleep Med, 2018. **45**: p. 74-79.
24. Okkerse, P., et al., *The use of a battery of pain models to detect analgesic properties of compounds: a two-part four-way crossover study.* Br J Clin Pharmacol, 2017. **83**(5): p. 976-990.
25. van de Loo, A., et al., *The effects of intranasal esketamine (84 mg) and oral mirtazapine (30 mg) on on-road driving performance: a double-blind, placebo-controlled study.* Psychopharmacology (Berl), 2017. **234**(21): p. 3175-3183.
26. Parrott, A.C. and I. Hindmarch, *Factor analysis of a sleep evaluation questionnaire.* Psychol Med, 1978. **8**(2): p. 325-9.
27. Borland, R.G. and A.N. Nicholson, *Visual motor co-ordination and dynamic visual acuity.* Br J Clin Pharmacol, 1984. **18 Suppl 1**: p. 69S-72S.
28. Steveninck, A.V., *Methods of assessment of central nervous system effects of drugs in man.* 1993, State University Leiden: Leiden.
29. Wright, B.M., *A simple mechanical ataxia-meter.* J Physiol, 1971. **218 Suppl**: p. 27P-28P.
30. van Steveninck, A.L., et al., *Pharmacodynamic interactions of diazepam and intravenous alcohol at pseudo steady state.* Psychopharmacology (Berl), 1993. **110**(4): p. 471-8.
31. van Steveninck, A.L., et al., *A study of the effects of long-term use on individual sensitivity to temazepam and lorazepam in a clinical population.* Br J Clin Pharmacol, 1997. **44**(3): p. 267-75.
32. van Steveninck, A.L., et al., *A comparison of the sensitivities of adaptive tracking, eye movement analysis and visual analog lines to the effects of incremental doses of temazepam in healthy volunteers.* Clin Pharmacol Ther, 1991. **50**(2): p. 172-80.
33. Baloh, R.W., et al., *Quantitative measurement of saccade amplitude, duration, and velocity.* Neurology, 1975. **25**(11): p. 1065-70.
34. Bittencourt, P.R., et al., *Benzodiazepines impair smooth pursuit eye movements.* Br J Clin Pharmacol, 1983. **15**(2): p. 259-62.
35. Norris, H., *The action of sedatives on brain stem oculomotor systems in man.* Neuropharmacology, 1971. **10**(21): p. 181-91.
36. de Visser, S.J., et al., *Biomarkers for the effects of antipsychotic drugs in healthy volunteers.* Br J Clin Pharmacol, 2001. **51**(2): p. 119-32.
37. Bond A, L.M., *The use of analogue scales in rating subjective feelings.* Br J Med Psychol 1974. **47**: p. 211-218.
38. Jobert, M., et al., *Guidelines for the recording and evaluation of pharmaco-EEG data in man: the International Pharmaco-EEG Society (IPEG).* Neuropsychobiology, 2012. **66**(4): p. 201-20.
39. Steenbergen, P., et al., *A system for inducing concurrent tactile and nociceptive sensations at the same site using electrocutaneous stimulation.* Behav Res Methods, 2012. **44**(4): p. 924-33.
40. Doll, R.J., et al., *Tracking of nociceptive thresholds using adaptive psychophysical methods.* Behav Res Methods, 2014. **46**(1): p. 55-66.
41. Mouraux, A., G.D. Iannetti, and L. Plaghki, *Low intensity intra-epidermal electrical stimulation can activate Adelta-nociceptors selectively.* Pain, 2010. **150**(1): p. 199-207.
42. van der Heide, E.M., et al., *Single pulse and pulse train modulation of cutaneous electrical stimulation: a comparison of methods.* J Clin Neurophysiol, 2009. **26**(1): p. 54-60.
43. Mouraux, A., E. Marot, and V. Legrain, *Short trains of intra-epidermal electrical stimulation to elicit reliable behavioral and electrophysiological responses to the selective activation of nociceptors in humans.* Neurosci Lett, 2014. **561**: p. 69-73.
44. Liang, M., et al., *Brain potentials evoked by intraepidermal electrical stimuli reflect the central sensitization of nociceptive pathways.* J Neurophysiol, 2016. **116**(2): p. 286-95.
45. Tzabazis, A.Z., et al., *Selective nociceptor activation in volunteers by infrared diode laser.* Mol Pain, 2011. **7**: p. 18.

46. Domnick, C., et al., *C-fiber-related EEG-oscillations induced by laser radiant heat stimulation of capsaicin-treated skin.* J Pain Res, 2009. **2**: p. 49-56.
47. Hullemann, P., et al., *Peripheral sensitization reduces laser-evoked potential habituation.* Neurophysiol Clin, 2015. **45**(6): p. 457-67.
48. Valeriani, M., et al., *Clinical usefulness of laser evoked potentials.* Neurophysiol Clin, 2012. **42**(5): p. 345-53.
49. Olofsen, E., et al., *Alfentanil and placebo analgesia: no sex differences detected in models of experimental pain.* Anesthesiology, 2005. **103**(1): p. 130-9.
50. Arendt-Nielsen, L., et al., *Effects of Gabapentin on Experimental Somatic Pain and Temporal Summation.* Reg Anesth Pain Med, 2007. **32**(5): p. 382-388.
51. Polianskis, R., T. Graven-Nielsen, and L. Arendt-Nielsen, *Pressure-pain function in desensitized and hypersensitized muscle and skin assessed by cuff algometry.* J Pain, 2002. **3**(1): p. 28-37.
52. Polianskis, R., T. Graven-Nielsen, and L. Arendt-Nielsen, *Computer-controlled pneumatic pressure algometry--a new technique for quantitative sensory testing.* Eur J Pain, 2001. **5**(3): p. 267-77.
53. Eckhardt, K., et al., *Same incidence of adverse drug events after codeine administration irrespective of the genetically determined differences in morphine formation.* Pain, 1998. **76**(1-2): p. 27-33.
54. Jones, S.F., et al., *Morphine and ibuprofen compared using the cold pressor test.* Pain, 1988. **34**(2): p. 117-22.
55. Van der Kloot, W.A., *De MPQ-DLV –een standaard Nederlandstalige versie van de McGill Pain Questionnaire*. 1990, Swets & Zeitlinger: Lisse
56. Verkes, R.J., W.A. Van der Kloot, and J. Van der Meij, *The perceived structure of 176 pain descriptive words.* Pain, 1989. **38**(2): p. 219-29.
57. Melzack, R., *The McGill Pain Questionnaire: major properties and scoring methods.* Pain, 1975. **1**(3): p. 277-99.

Appendix 1. Perceived driving quality scale (English version).

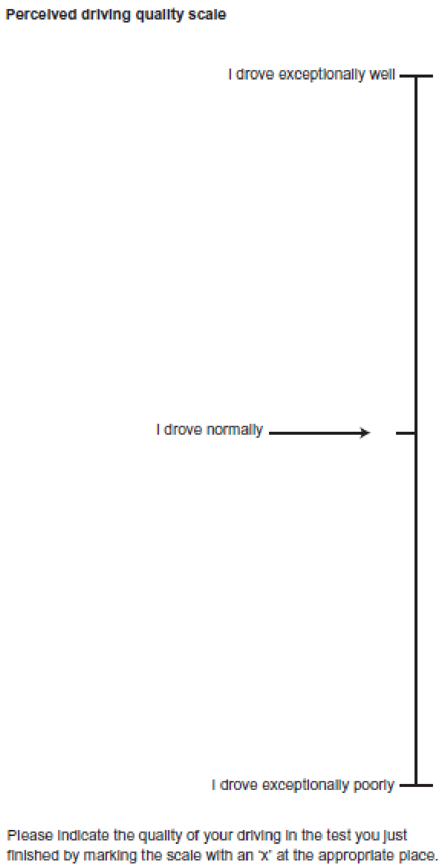

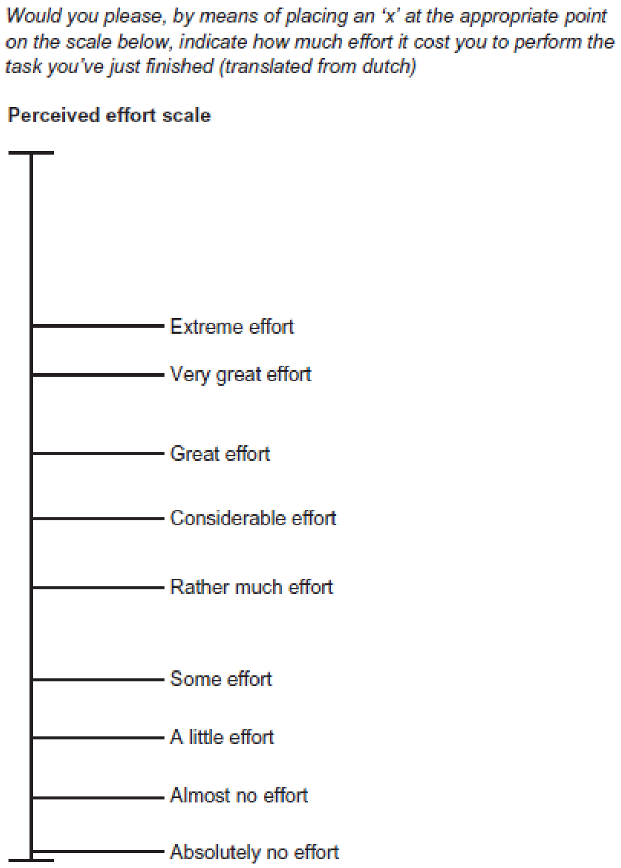

Appendix 2. Perceived driving quality scale (Dutch version).

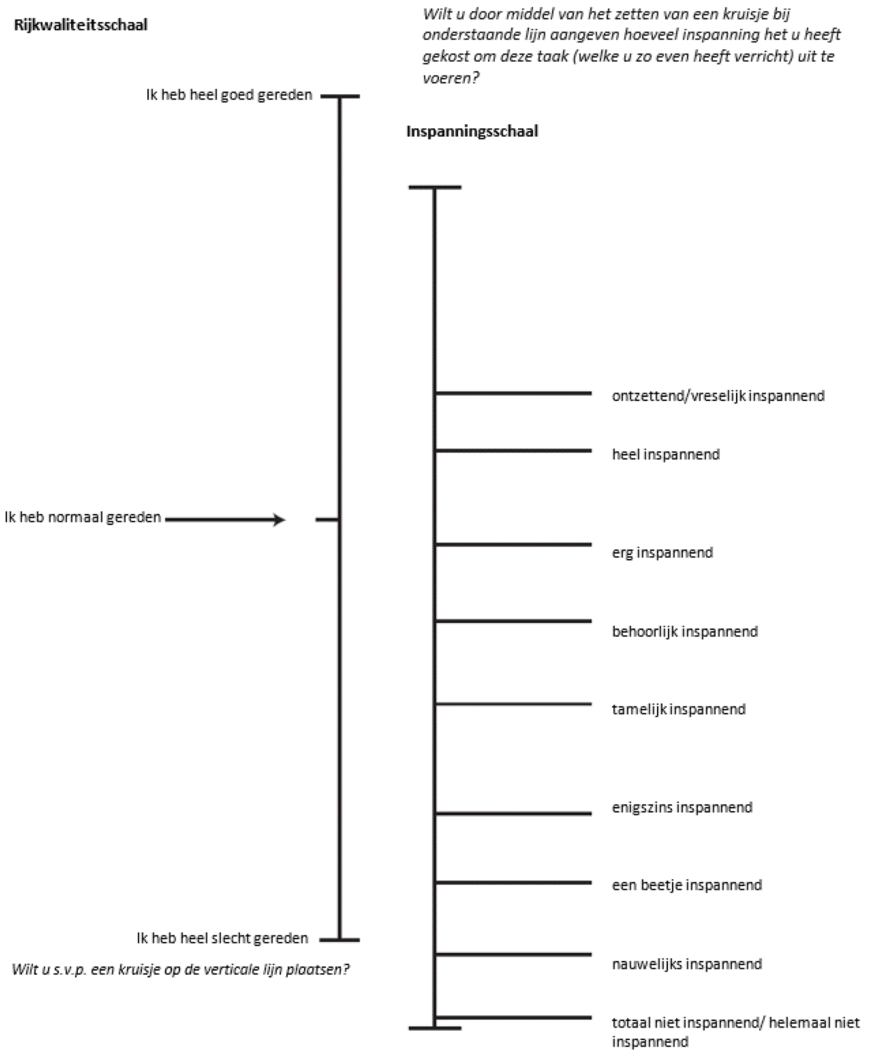

Appendix 3. Instructor assessment of driving quality (English version).

Action	Mark (1-10) or N/A[1]	Comments
Visual Scanning		
Changing gear		
Steering		
Braking		
Clutch		
Speed		
Corners		
Anticipate/Insight		
Apply traffic rule		
Focus		
Reaction time		

[1] N/A: Not Applicable.

Appendix 4. Instructor assessment of driving quality (Dutch).

**Action**	**Score (1-10) of Nvt[1]**	**Opmerkingen**
Visueel scannen		
Schakelen		
Sturen		
Remen		
Koppelen		
Snelheid		
Hoeken		
Anticiperen & inzicht		
Toepassen verkeersregels		
Aandacht		
Reactie tijd		

[1] Nvt: Niet van toepassing.

Appendix 5. Karolinska Sleepiness Scale (English version).

Please, indicate your sleepiness during the 5 minutes before this rating through circling the appropriate description below. *Use also the intermediate steps!*

1 = very alert

2

3 = alert - normal level

4

5 = neither alert nor sleepy

6

7 = sleepy, but no effort to keep awake

8

9 = very sleepy, great effort to keep awake, fighting sleep

Appendix 6. Karolinska Sleepiness Scale (Dutch version).

Geef uw slaperigheid gedurende de 5 minuten voor deze beoordeling aan door de meest passende beschrijving te omcirkelen. *Gebruik ook de tussenliggende stappen!*

1 = heel erg alert

2

3 = alert - normaal niveau

4

5 = niet alert en niet slaperig

6

7 = slaperig, maar geen moeite om wakker te blijven

8

9 = erg slaperig, grote moeite om wakker te blijven, vechten tegen de slaap

Appendix 7. Leeds Sleep Evaluation Questionnaire (English Version).

How would you compare getting to sleep using the medication with getting to sleep normally, i.e. without medication?

1	Harder than usual	______________	Easier than usual	**GTS**<br>**Getting to sleep**
2	Slower than usual	______________	Quicker than usual	
3	Felt less drowsy than usual	______________	Felt more drowsy than usual	

How would you compare the quality of sleep using the medication with non-medicated (your usual) sleep?

4	More restless than usual	______________	More restful than usual	**QOS**<br>**Quality of sleep**
5	More periods of wakefulness than usual	______________	Fewer periods of wakefulness than usual	

How did your awakening after medication compare with your usual pattern of awakening?

6	More difficult than usual	______________	Easier than usual	**AFS**<br>**Awake following sleep**
7	Took longer than usual	______________	Shorter than usual	

How did you feel on waking?

8	Tired	______________	Alert	**BFW**<br>**Behaviour following wakening**

How do you feel now?

9	Tired	______________	Alert

How would you describe your balance and co-ordination upon awakening?

10	More clumsy than usual	______________	Less clumsy than usual

Appendix 8. Leeds Sleep Evaluation Questionnaire (Dutch Version).

Hoe zou u het in slaap vallen met het onderzoeksmiddel vergelijken met normaal in slaap vallen, d.w.z. zonder het onderzoeksmiddel?

1	Gemakkelijker dan gewoonlijk	______	Moeilijker dan gewoonlijk	
2	Langzamer dan gewoonlijk	______	Sneller dan gewoonlijk	**GTS** Inslapen
3	Ik voelde me slaperiger dan gewoonlijk	______	Ik voelde me minder slaperig dan gewoonlijk	

Hoe zou u de kwaliteit van het slapen met het onderzoeksmiddel vergelijken met uw normale slaap, d.w.z. zonder het onderzoeksmiddel?

4	Onrustiger dan gewoonlijk	______	Rustiger dan gewoonlijk	**QOS**
5	Meer periodes van wakker liggen dan gewoonlijk	______	Minder periodes van wakker liggen dan gewoonlijk	Kwaliteit van slaap

Hoe was het wakker worden na het onderzoeksmiddel vergeleken met uw gebruikelijke patroon van wakker worden?

6	Moeilijker dan gewoonlijk	______	Makkelijker dan gewoonlijk	**AFS**
7	Het duurde langer dan gewoonlijk	______	Het duurde korter dan gewoonlijk	Ontwaken

Hoe voelde u zich bij het wakker worden?

8	Vermoeid	______	Alert	

Hoe voelt u zich nou?

9	Vermoeid	______	Alert	**BFW** Gedrag na ontwaken

Hoe was uw evenwichts- en coördinatiegevoel bij het opstaan?

10	Onhandiger dan gewoonlijk	______	Minder onhandig dan gewoonlijk	

Appendix 9. Visual analogue scale Bond-Lader (English version).

(English original version)

(As described in : Bond A, Lader M. The use of analogue scales in rating subjective feelings. Br J Med Psychol 1974;47:211-18)

1. Please rate the way you feel in terms of the dimensions given below.
2. Regard the line as representing the full range of each dimension.
3. Rate your feelings as they are at the moment.
4. Mark clearly and perpendicularly across each line.

1 Alert ____ Drowsy

2 Calm ____ Excited

3 Strong ____ Feeble

4 Confused ____ Clear-headed

5 Well-coordinated ____ Clumsy

6 Lethargic ____ Energetic

7 Contented ____ Discontented

8 Troubled ____ Tranquil

9 Mentally slow ____ Quick-witted

10 Tense ____ Relaxed

11 Attentive ____ Dreamy

12 Incompetent ____ Proficient

13 Happy ____ Sad

14 Antagonistic ____ Amicable

15 Interested ____ Bored

16 Withdrawn ____ Gregarious

(*Line numbers refer to VAS-item definition in the ProMaSys study database)

Appendix 10. Visual analogue scale Bond-Lader (Dutch version).

(Dutch translated version)

(Dutch version of visual analogue scales of alertness, mood and calmness, as described in : Bond A, Lader M. The use of analogue scales in rating subjective feelings.
Br J Med Psychol 1974;47:211-18)

Op de onderstaande lijnen kunt U met een streepje aangeven hoe U zich op dit moment voelt. Aan de uiteinden van de lijnen staan twee uitersten van een bepaald gevoel. Door de plaats van het streepje op de horizontale lijn te kiezen kunt U aangeven hoe U zich voelt. Een streep door het uiteinde van de lijn geeft aan dat U het gevoel in zeer sterke mate zo ervaart, een streep door het midden van de lijn geeft aan dat Uw gevoel het midden houdt tussen twee uitersten etc.

1 Alert ____ Suf

2 Kalm ____ Opgewonden

3 Sterk ____ Zwak

4 Warrig ____ Helder

5 Handig ____ Onhandig

6 Slaperig ____ Energiek

7 Tevreden ____ Ontevreden

8 Verstoord ____ Rustig

9 Traag ____ Scherp van geest

10 Gespannen ____ Ontspannen

11 Oplettend ____ Dromerig

12 Incompetent ____ Bekwaam

13 Gelukkig ____ Ongelukkig

14 Recalcitrant ____ Vriendschappelijk

15 Geinteresseerd ____ Verveeld

16 Teruggetrokken ____ Sociaal

(*Line numbers refer to VAS-item definition in the ProMaSys study database)

Appendix 11. McGill Pain Questionnaire Short Version (SF-MPQ) (English version).

A. DESCRIBE YOUR PAIN *(choose one option on each row.)*

	None	Mild	Moderate	Severe
1. Throbbing	0 ☐	1 ☐	2 ☐	3 ☐
2. Shooting	0 ☐	1 ☐	2 ☐	3 ☐
3. Stabbing	0 ☐	1 ☐	2 ☐	3 ☐
4. Sharp	0 ☐	1 ☐	2 ☐	3 ☐
5. Cramping	0 ☐	1 ☐	2 ☐	3 ☐
6. Gnawing	0 ☐	1 ☐	2 ☐	3 ☐
7. Hot burning	0 ☐	1 ☐	2 ☐	3 ☐
8. Aching	0 ☐	1 ☐	2 ☐	3 ☐
9. Heavy	0 ☐	1 ☐	2 ☐	3 ☐
10. Tender	0 ☐	1 ☐	2 ☐	3 ☐
11. Splitting	0 ☐	1 ☐	2 ☐	3 ☐
12. Tiring Exhausting	0 ☐	1 ☐	2 ☐	3 ☐
13. Sickening	0 ☐	1 ☐	2 ☐	3 ☐
14. Fearful	0 ☐	1 ☐	2 ☐	3 ☐
15. Punishing cruel	0 ☐	1 ☐	2 ☐	3 ☐

B. SCORE YOUR PAIN

The line below indicates he severity of the pain and runs from "no pain" to "worst possible pain". Place on the line a vertical dash (|) on the place that indicates your pain score.

No pain — **Worst possible pain**

☐☐☐

Score in mm
(Investigator's use only)

C. HUIDIGE INTENSITEIT VAN DE PIJN

0 ☐ No pain

1 ☐ Mild

2 ☐ Discomforting

3 ☐ Distressing

4 ☐ Horrible

5 ☐ Excruciating

Appendix 12. McGill Pain Questionnaire Short Version (SF-MPQ) (Dutch version).

A. BESCHRIJF UW PIJN *(één hokje aankruisen op elke regel.)*

	Geen	Licht	Matig	Ernstig
1. Kloppend	0 ☐	1 ☐	2 ☐	3 ☐
2. In scheuten	0 ☐	1 ☐	2 ☐	3 ☐
3. Stekend	0 ☐	1 ☐	2 ☐	3 ☐
4. Scherp	0 ☐	1 ☐	2 ☐	3 ☐
5. Krampend	0 ☐	1 ☐	2 ☐	3 ☐
6. Knagend	0 ☐	1 ☐	2 ☐	3 ☐
7. Warm-brandend	0 ☐	1 ☐	2 ☐	3 ☐
8. Zeurend	0 ☐	1 ☐	2 ☐	3 ☐
9. Drukkend	0 ☐	1 ☐	2 ☐	3 ☐
10. Gevoelig	0 ☐	1 ☐	2 ☐	3 ☐
11. Splijtend	0 ☐	1 ☐	2 ☐	3 ☐
12. Vermoeiend-uitputtend	0 ☐	1 ☐	2 ☐	3 ☐
13. Misselijk makend	0 ☐	1 ☐	2 ☐	3 ☐
14. Verontrustend	0 ☐	1 ☐	2 ☐	3 ☐
15. Wreed-gemeen	0 ☐	1 ☐	2 ☐	3 ☐

B. BEOORDEEL UW PIJN

De onderstaande lijn geeft de hevigheid van de pijn aan en loopt van “geen pijn” tot “ ondraaglijke pijn”. Zet op de lijn een verticaal streepje (I) op de plek die het beste uw pijn aangeeft.

Geen Pijn —————————————— **Ondraaglijk pijn**

☐☐☐ Score in mm

(Investigator's use only)

C. HUIDIGE INTENSITEIT VAN DE PIJN

0 ☐ Geen pijn

1 ☐ Licht

2 ☐ Matig

3 ☐ Hevig

4 ☐ Zeer hevig

5 ☐ Ondraaglijk
